# Sustainable L2 writing pedagogy in Turkish higher education: Effects of AI-mediated feedback on self-regulated learning and writing performance

**DOI:** 10.1371/journal.pone.0344618

**Published:** 2026-07-14

**Authors:** Ahmet Erkam Selvi, Aydan Irgatoğlu, Oğuz Yurttadur, Gürkan Dağbaşı

**Affiliations:** 1 Department of Arabic Language Teaching, Gazi University, Ankara, Türkiye; 2 School of Foreign Languages, Ankara Hacı Bayram Veli University, Ankara, Türkiye; 3 Department of Sculpture, Selçuk University, Konya, Türkiye; Public Library of Science, UNITED KINGDOM OF GREAT BRITAIN AND NORTHERN IRELAND

## Abstract

In response to growing demands for sustainable, scalable, and learner-centered feedback practices in higher education, this study examined the effects of different feedback modalities on Foreign Language (FL) learners’ self-regulated learning (SRL) strategies and writing performance within the Turkish university context. Guided by self-regulated learning theory and national digital transformation priorities, a quasi-experimental design was employed with three groups: an Automated Feedback (AF) group using Grammarly, a Generative AI Feedback (GenAI-F) group using ChatGPT, and an Instructor-Mediated Feedback (IMF) group without automated feedback. Eighty-four preparatory school students participated in a ten-week intervention involving multiple argumentative writing tasks, pre- and post-writing assessments, and a validated SRL strategy questionnaire. Mixed factorial ANOVAs revealed significant main effects of time on cognitive and metacognitive SRL strategies, alongside significant interaction effects between feedback type and time for idea planning and interest enhancement. The GenAI-F group demonstrated the largest gains in metacognitive and motivational regulation strategies and achieved significantly higher improvements in overall writing performance and content quality compared with the AF and IMF groups. Moderation analyses further indicated that CEFR-based proficiency levels shaped feedback effectiveness, with GenAI-F yielding stronger benefits for intermediate-level learners. These findings suggest that generative AI-mediated feedback can support sustainable EFL writing instruction by fostering autonomous learning capacities, with potential implications for reducing instructor workload through scalable feedback support, although this dimension was not directly measured in the present study, and promoting lifelong learning skills aligned with higher education sustainability agendas.

## Introduction

Effective feedback is central to the development of academic writing proficiency and learner autonomy in second and foreign language (L2) education. In foreign language learning contexts such as Türkiye, where large class sizes and limited instructional time constrain opportunities for individualized instructor feedback, the need for sustainable, equitable, and scalable feedback practices has become increasingly evident [1,2]. These contextual challenges have intensified interest in technology-enhanced feedback solutions capable of supporting writing development while reducing instructional burden.

Recent advances in artificial intelligence (AI) have introduced new possibilities for addressing such structural constraints through automated and generative feedback systems, fundamentally reshaping how feedback is delivered, interpreted, and utilized in higher education. Among AI-mediated feedback tools, automated feedback (AF) systems such as Grammarly and generative AI feedback (GenAI-F) tools such as ChatGPT represent two distinct approaches with differing pedagogical affordances. AF systems primarily target form-focused aspects of writing, including grammar, vocabulary, and mechanics, relying on predefined algorithms and scoring criteria [3]. Research conducted in L2 contexts, including Türkiye, suggests that AF can support linguistic accuracy and reduce surface-level errors. While earlier research has often characterized automated feedback systems as primarily effective for form-focused aspects of writing, more recent evidence points to a broader impact. A multi-level meta-analysis reports a moderate overall effect of automated feedback on students’ writing performance (g = 0.55), suggesting that its influence may extend beyond surface-level correction. In addition, experimental findings indicate that automated feedback can support key dimensions of self-regulated learning, including goal setting, planning, time management, as well as learners’ self-efficacy and satisfaction [4,5]. At the same time, advances in AI-driven feedback, including more adaptive and context-sensitive systems, have begun to extend support to certain higher-order writing concerns such as content development and organization [6,7]. However, the extent to which such improvements reflect deeper metacognitive engagement and sustained development in higher-order writing remains an open question. In particular, while AI-assisted feedback may offer expanded affordances for revision, it may provide more limited opportunities for dialogic reflection, strategic decision-making, and the development of independent evaluative judgment. The present study builds on this evolving body of research by comparatively examining how different feedback modalities shape both writing performance and self-regulated learning strategies within a sustainability-oriented framework.

In contrast, recent developments in generative AI have expanded the scope of AI-mediated feedback by enabling context-sensitive, discursive, and explanatory responses. Unlike conventional AF systems, GenAI-F tools can provide feedback on content, coherence, organization, and rhetorical effectiveness while engaging learners in interactive and reflective dialogue [8,9]. Emerging empirical evidence indicates that GenAI-F can facilitate idea generation, encourage deeper revision practices, and promote learner autonomy in academic writing [10,11]. Within the Turkish L2 context, where students often rely heavily on instructor authority and model texts, such dialogic feedback may offer more inclusive and sustainable writing support by fostering independent learning pathways.

However, feedback effectiveness cannot be examined independently of learner-related factors, particularly language proficiency. Previous research consistently demonstrates that learners’ uptake and use of feedback are mediated by their linguistic and cognitive resources [12,13]. Studies conducted in Turkish higher education contexts similarly report that learners with lower proficiency levels often struggle to interpret indirect or metalinguistic feedback, limiting its impact on revision quality [14]. Despite growing interest in AI-mediated feedback, empirical evidence remains limited regarding how different feedback types interact with proficiency levels to influence writing development in Türkiye.

Beyond immediate writing performance, feedback also plays a critical role in fostering self-regulated learning (SRL), a cornerstone of sustainable and lifelong education. Grounded in sociocognitive theory, SRL refers to learners’ proactive capacity to plan, monitor, and evaluate their learning processes through the dynamic interaction of cognition, behavior, and learning environments [15,16]. In L2 writing, SRL is particularly important given the sustained cognitive effort, strategic decision making, and emotional regulation required throughout the writing process.

Research conceptualizes SRL in L2 writing as a multidimensional construct encompassing cognitive, metacognitive, motivational, and social strategies [17]. Cognitive strategies support text generation and linguistic processing, while metacognitive strategies enable goal setting, planning, and self-monitoring. Motivational regulation strategies help sustain effort and engagement, and social strategies facilitate help-seeking and interaction with feedback sources. Empirical studies consistently demonstrate that learners who actively employ a wide range of SRL strategies produce higher-quality writing and exhibit greater autonomy in academic tasks [18,19]. From a sustainability perspective, SRL represents a transferable competence that supports learner agency beyond formal educational settings.

Feedback functions as a central catalyst for the development of SRL by providing information about performance relative to learning goals and enabling learners to regulate subsequent learning behaviors [20]. Within SRL frameworks, feedback operates as part of an iterative feedback loop in which learners interpret external input, generate internal feedback, and adjust strategies accordingly [16]. Recent research has begun to conceptualize AI-supported writing environments as metacognitive scaffolding systems that actively shape learners’ self-regulatory processes rather than merely providing corrective feedback. Studies examining learners’ engagement with AI-generated feedback suggest that such interaction can enhance metacognitive awareness by requiring learners to interpret, evaluate, and selectively incorporate feedback into their revisions [21,22]. This interaction fosters recursive cycles of planning, monitoring, and evaluation, which are central to self-regulated learning. Empirical findings further indicate that learners who engage critically with feedback tend to produce more coherent and rhetorically effective texts [22]. In addition, emerging research on generative AI tools highlights their role as dialogic partners that support reflective thinking and strategy regulation during writing [23,24]. However, these studies also emphasize that metacognitive development is not automatic; its effectiveness depends on learners’ ability to regulate their interaction with AI tools, including questioning feedback, cross-checking suggestions, and maintaining authorial control [11]. Taken together, this body of research suggests that generative AI feedback can facilitate deeper forms of self-regulation by externalizing metacognitive processes and making them more visible and actionable during writing. In L2 writing, effective feedback supports not only error correction but also reflection, self-evaluation, and strategic engagement with writing tasks [24]. While instructor-mediated feedback has been shown to promote SRL development, practical constraints in higher education limit the sustainability of individualized feedback practices, particularly in large classes.

AI-mediated feedback has therefore attracted increasing attention as a scalable alternative capable of supporting continuous learner engagement. Automated feedback systems have been shown to enhance linguistic accuracy and writing fluency, particularly in form-focused dimensions [25,26], yet they often provide limited support for meaning-level revisions or individualized strategy development [27]. Generative AI feedback tools, by contrast, offer interactive and adaptive feedback that may better support metacognitive awareness, reflection, and strategic writing behaviors [8,11]. Despite this potential, comparative research examining how AF and GenAI-F differentially influence writing performance and SRL strategy use remains scarce, particularly in non-Western higher education contexts.

Furthermore, the moderating role of language proficiency in AI-mediated feedback contexts has received insufficient empirical attention. While higher-proficiency learners tend to engage more selectively and critically with feedback, integrating it into their existing linguistic repertoires, lower-proficiency learners may experience cognitive overload when confronted with extensive corrective input [12,28]. Preliminary evidence suggests that GenAI-F may offer adaptive scaffolding through explanation and dialogue, potentially mitigating proficiency-related barriers [9]. Yet, this assumption remains underexplored in Turkish L2 writing contexts.

Addressing these gaps, the present study investigates the effects of two forms of AI-mediated feedback, automated feedback and generative AI feedback, on Turkish L2 learners’ writing performance and use of self-regulated learning strategies, while also examining the moderating role of English language proficiency as operationalized through CEFR levels. Situated within a Turkish state university context, this research aims to contribute to ongoing discussions on sustainable, technology-enhanced writing pedagogy and to inform pedagogical and policy-oriented decision making in support of inclusive and high-quality language education. Although prior research has established functional differences between automated feedback and generative AI feedback, particularly in relation to form-focused versus meaning-level support, several important gaps remain. First, much of the existing literature has examined these tools in isolation rather than within a comparative instructional framework that reflects authentic classroom conditions. Second, limited attention has been paid to how different feedback modalities shape learners’ self-regulated learning processes, including metacognitive and motivational strategy use, which are central to sustainable writing development. Third, the interaction between feedback type and learner characteristics, such as language proficiency, remains underexplored, particularly in non-Western higher education contexts. As a result, while the affordances of AI tools are increasingly recognized, there is still insufficient empirical evidence regarding how these feedback modalities differentially influence both writing performance and self-regulated learning over time. Addressing these gaps provides the primary rationale for the present study. Building on these gaps, the present study contributes to the literature in several important ways. It provides a direct comparison of automated feedback, generative AI feedback, and instructor-mediated feedback within an authentic classroom context, offering ecologically valid evidence of their relative effectiveness. In addition, it adopts a multidimensional perspective by examining not only writing performance but also the development of self-regulated learning strategies, thereby shedding light on the mechanisms through which feedback influences learning. Finally, by incorporating proficiency as a moderating variable, the study offers a more nuanced understanding of how different learners benefit from AI-mediated feedback, contributing to more equitable and sustainable pedagogical practices. In this study, development is operationalized as change over time for Research Questions 1 and 2, whereas Research Question 3 focuses on post-intervention differences to examine how proficiency moderates the effects of feedback type on learning outcomes. Specifically, the study addresses the following research questions:

1. To what extent do automated feedback (AF), generative AI feedback (GenAI-F), and instructor-mediated feedback (IMF) differentially influence changes over time in Turkish L2 learners’ self-regulated learning strategies in academic writing?
2. To what extent do automated feedback (AF), generative AI feedback (GenAI-F), and instructor-mediated feedback (IMF) differentially influence changes over time in Turkish L2 learners’ writing performance?
3. To what extent does L2 proficiency moderate the effects of feedback type on learners’ post-test writing performance and self-regulated learning outcomes?

## Materials and methods

### Research context

The study was conducted in a credit-bearing compulsory English course offered to preparatory school undergraduate students from non-English majors at a public state university in Türkiye. The course aimed to develop students’ integrated academic English skills, including reading, listening, speaking, and writing, in alignment with the learning outcomes specified by the Council of Higher Education (YÖK). Instruction was delivered in two 90-minute sessions per week over a 14-week semester.

Similar to many Turkish higher education contexts, the course was characterized by large class sizes and intensive curricular demands, which limited instructors’ ability to provide individualized, process-oriented feedback on student writing. Consequently, writing tasks were typically evaluated using holistic rubrics aligned with CEFR descriptors, with limited formative commentary, a situation that reflects a broader need for sustainable and scalable feedback practices in Turkish L2 instruction [2,29].

### Research design and participants

Participants were recruited through convenience sampling from three intact classes taught by the same instructor to minimize instructional variability. A total of 86 students (58 females, 28 males) voluntarily participated in the study. The classes were randomly assigned to one of three conditions: a IMF group receiving no AI-mediated feedback (n = 27), an AF feedback group using Grammarly (n = 29), and a GenAI-F group using ChatGPT (n = 30). Participants’ ages ranged from 18 to 22 years (M = 18.9, SD = 0.74). All participants had studied English for approximately 11 years in the Turkish education system and reported no long-term overseas study experience.

English proficiency was measured using the Standard English Test (SET) and interpreted according to CEFR benchmarks. CEFR level classifications were determined based on the official score equivalency guidelines provided by the EF Standard English Test. Specifically, SET scores ranging from 41–60 were categorized as B1–B2 (intermediate), while scores within the range of 61–70 were classified as B2–C1 (advanced). These thresholds are aligned with standardized CEFR descriptors and have been widely used in research and institutional placement contexts. To ensure consistency, all participants’ scores were converted into CEFR levels prior to analysis, and grouping decisions were verified against institutional proficiency benchmarks used within the preparatory program. In cases where scores fell at boundary thresholds, classification decisions were cross-checked with CEFR descriptor profiles to ensure appropriate level assignment. Based on their scores, students were categorized into intermediate-level learners (B1–B2; n = 40) and advanced-level learners (B2–C1; n = 44). Two students did not report valid scores and were excluded from proficiency-based analyses. One-way ANOVA confirmed no statistically significant differences in proficiency across the three groups at baseline (p > .05). A chi-square test further indicated that proficiency level was not significantly associated with group assignment, supporting the homogeneity of the groups.

The participant recruitment process for this study began on February 3, 2025, and ended on May 9, 2025. Prior to participation, all students were informed about the research purpose, procedures, voluntary nature of participation, and their right to withdraw at any stage without academic penalty. This study was conducted in accordance with the ethical standards of the Declaration of Helsinki. Ethical approval for the research was obtained from the Scientific Research and Publication Ethics Committee of Ankara Hacı Bayram Veli University. The committee reviewed and approved the study protocol, including the data collection procedures and instruments. Participation in the study was voluntary. Prior to data collection, all participants were informed about the purpose of the research, the nature of their involvement, and their rights as participants. Informed consent was obtained from all participants before participation. No personally identifiable information was collected, and all responses were recorded anonymously. Participants were informed that they could withdraw from the study at any time without any consequences. The collected data were used solely for academic research purposes and were stored securely in accordance with institutional data protection guidelines.

### Data collection

Data were collected over a ten-week period within the 14-week semester, following an initial two-week instructional phase and preceding the final two weeks of the course. The initial phase was devoted to course orientation, baseline instruction, and administration of the proficiency test and pre-test measures. The ten-week data collection period constituted the main intervention phase, during which students completed three writing tasks under their assigned feedback conditions. The final two weeks of the semester were reserved for post-test administration and course completion activities. This study did not involve retrospective analysis of archived samples, or previously collected datasets. All data were collected prospectively during the recruitment period (February 3, 2025 – May 9, 2025) specifically for the purposes of this research. The authors did not have access to personally identifiable information during or after data collection. All data were anonymized prior to analysis, and participants were assigned coded identifiers.

All participants completed three independent argumentative writing tasks integrated into the course syllabus. The tasks did not involve source-based or integrated writing; rather, students were required to generate and develop their own ideas in response to a given prompt without access to external materials. Each task was designed to elicit structured argumentation, including the presentation of a clear thesis, supporting arguments, and relevant examples. The prompts were aligned with course themes and CEFR B1–B2 writing descriptors. For example, one task required students to respond to the prompt: “Do you think online education is more effective than face-to-face education? Discuss your opinion and provide reasons and examples.” Similar prompts addressed familiar academic topics such as technology use, social media, and environmental issues, ensuring comparability in cognitive and linguistic demands across tasks. All writing tasks were completed under controlled classroom conditions within 30 minutes and without access to dictionaries or digital resources. Task difficulty and topic familiarity were kept consistent across writing sessions to minimize variability unrelated to the experimental conditions.

To minimize potential cross-contamination between experimental conditions, specific procedural controls were implemented throughout the intervention. All in-class writing tasks were completed under controlled classroom conditions without access to external digital tools, including AI-based applications. Students were explicitly instructed not to use external feedback tools, including Grammarly and generative AI systems such as ChatGPT, for the assigned writing tasks. To monitor compliance, a brief post-intervention self-report check was administered, in which participants were asked to indicate whether they had used any external feedback tools during the intervention period. The responses indicated no systematic or significant use of unauthorized AI tools within the IMF group, and no evidence of cross-group contamination that would threaten the internal validity of the study. While the possibility of unreported tool use cannot be entirely excluded in naturalistic educational settings, the combination of controlled task conditions, explicit instructions, and post-hoc verification procedures provides reasonable assurance that the observed differences between groups reflect the intended feedback interventions. For each task, students wrote a 150–180-word essay within 30 minutes under controlled classroom conditions and without access to external resources. Writing prompts were aligned with course themes and instructional objectives.

Students in the instructor-mediated feedback (IMF) group followed the standard instructional practices of the course, which reflect common feedback conditions in Turkish higher education contexts characterized by large class sizes and time constraints. After completing each writing task, students received holistic scores based on a CEFR-aligned analytic rubric assessing content, organization, vocabulary, language use, and mechanics. In addition to these scores, the instructor provided brief whole-class oral feedback addressing common strengths and weaknesses observed across student texts, such as frequent grammatical errors, issues in paragraph organization, and limitations in idea development. However, no individualized written feedback, marginal comments, or text-specific corrective annotations were provided, and students were not required to revise or resubmit their drafts. Feedback was delivered once per task, typically during the subsequent class session, and was designed to mirror prevalent assessment-oriented practices in exam-driven EFL contexts in Türkiye. This condition therefore represents a baseline instructional model characterized by limited formative feedback and minimal opportunities for iterative revision, consistent with prior research documenting feedback constraints in large-scale L2 writing classrooms (e.g., studies on feedback practices and scalability in EFL contexts). The IMF condition was intentionally structured to provide a pedagogically realistic comparison group, allowing the effects of AI-mediated feedback to be interpreted relative to commonly implemented instructor-led feedback practices rather than an artificially enhanced control condition. Students in the automated feedback (AF) group revised their drafts using feedback generated by Grammarly, which provides algorithm-based feedback on grammar, vocabulary use, clarity, and mechanics. Students in the generative AI feedback (GenAI-F) group revised their drafts using ChatGPT, which was employed solely as a feedback provider. These students were instructed to submit their drafts and request rubric-based feedback addressing content, organization, language use, and coherence. To support consistent implementation across conditions, students in the AF and GenAI-F groups received structured training on tool use, including demonstration of key functions (e.g., grammar correction, prompt formulation, and interpretation of feedback) and guided practice during initial sessions. During in-class writing and revision, use of the assigned tool was monitored by the instructor, and students were required to complete the tasks under controlled classroom conditions without access to alternative tools. In addition, brief post-task checklists were used to confirm that students had engaged with the assigned feedback (e.g., whether revisions were made based on tool suggestions and which features were used). While these procedures were intended to standardize exposure and encourage active use, the study did not capture fine-grained usage data (e.g., logs of interaction frequency, time-on-task, or depth of engagement). Therefore, variability in individual engagement with the tools cannot be fully ruled out.

To ensure ethical and pedagogically appropriate use of AI tools, students in both experimental groups participated in structured 20-minute training sessions prior to each writing task. The training focused on understanding the scope and limitations of AI-mediated feedback, generating rubric-aligned prompts, critically evaluating feedback suggestions, and integrating feedback selectively. Sample prompts were provided, and students were encouraged to adapt them as needed. To ensure equity, students in the IMF group were granted access to the same training materials and AI tools after the completion of the study. To minimize potential methodological bias arising from the dual role of generative AI as both an instructional tool and an assessment aid, the scoring process was strictly standardized through rubric-based prompts, and human validation procedures were incorporated. In addition, scoring was conducted independently of the instructional phase, and no iterative feedback loops between scoring outputs and student revisions were allowed.

### Data collection tools

Three instruments were used for data collection. To assess changes in writing performance, all participants completed a pre-test and a post-test argumentative writing task under examination conditions. Each test required a minimum of 150 words within 30 minutes. Essays were evaluated using the L2 Composition Profile [30], which assesses content, organization, vocabulary, language use, and mechanics. Initial scoring was conducted using ChatGPT-4 with rubric-embedded prompts to ensure consistency. In this study, ChatGPT-4 was not treated as an independent scoring instrument but as a rubric-guided evaluation assistant. All scoring prompts were explicitly aligned with the L2 Composition Profile criteria to ensure consistency with established assessment standards. To enhance scoring validity, a subset of 30% of the essays was independently rated by an experienced human instructor, and interrater reliability analysis demonstrated a strong level of agreement (ICC = 0.81). This procedure is consistent with emerging research practices that use AI-assisted scoring in conjunction with human validation. However, it is acknowledged that the majority of scripts were scored by the AI-assisted procedure, which represents a methodological limitation. An experienced L2 writing instructor independently rated a randomly selected 30% of the essays. Interrater reliability between AI-assisted and human ratings was satisfactory, with an intraclass correlation coefficient of 0.81.

Students’ use of self-regulated learning strategies was measured using the Writing Strategies for Self-Regulated Learning Questionnaire (WSSRLQ) [17]. The 35-item instrument assessed four dimensions: cognitive, metacognitive, motivational regulation, and social-behavioral strategies. The questionnaire was translated into Turkish and back-translated to ensure linguistic equivalence. Responses were recorded on a seven-point Likert scale. Cronbach’s alpha values ranged from 0.75 to 0.84 across subscales, indicating satisfactory reliability.

English language proficiency was assessed using the EF Standard English Test, a validated online assessment aligned with CEFR descriptors [31]. Test scores were used to classify participants into proficiency groups for moderation analyses. At the beginning of the semester, participants completed the proficiency test, the pre-test writing task, and the SRL questionnaire. During the intervention, students completed three writing tasks under their assigned feedback conditions. At the end of the intervention period, all participants completed the post-test writing task and the SRL questionnaire within the same week. No system-generated usage logs were collected; engagement with feedback tools was monitored through in-class supervision and brief post-task self-reports.

### Data analysis

All quantitative analyses were conducted using SPSS 23. Preliminary checks confirmed normality, homogeneity of variance, and equivalence of covariance matrices. To address RQ1 and RQ2, A mixed-design ANOVA was selected as it allows examination of both within-subject changes over time (pre-test to post-test) and between-group differences across feedback conditions. For the moderation analysis (RQ3), a two-way ANOVA was conducted using post-test scores. This approach was selected to examine how proficiency level interacts with feedback type in shaping final learning outcomes, rather than changes over time. Effect sizes were reported using partial eta squared and Cohen’s d, with Bonferroni corrections applied for multiple comparisons. Although multiple dependent variables were analyzed, separate mixed-design ANOVAs were conducted for each outcome variable to allow dimension-specific interpretation of self-regulated learning strategies and writing subcomponents. This approach is consistent with prior research examining multidimensional SRL constructs, where individual components are theoretically distinct. To mitigate the risk of inflated Type I error associated with multiple comparisons, Bonferroni adjustments were applied within each family of post hoc tests. Nevertheless, the results should be interpreted with caution, and future studies may consider multivariate approaches (e.g., MANOVA/MANCOVA) to account for potential intercorrelations among dependent variables.

All relevant data are provided as Supporting Information files. The dataset (S1 File) includes anonymized participant-level data underlying all analyses reported in the study, including writing performance scores, subcomponents, group assignments, and self-regulated learning responses. A detailed codebook (S2 File), statistical analysis syntax (S3 File), R script (S4 File), and Structural Equation Modeling (SEM) script (S5 File) are also provided to ensure full reproducibility of the results.

## Results

This section presents the findings in relation to the research questions. Results are organized into three subsections addressing (1) self-regulated learning strategies, (2) writing performance, and (3) the moderating role of proficiency. Unless otherwise indicated, interpretations are based on statistically significant effects. Descriptive differences in the absence of statistical significance are reported cautiously and are not interpreted as evidence of differential impact.

The mixed factorial ANOVAs revealed statistically significant Time × Feedback Condition interaction effects for selected self-regulated learning (SRL) strategies. Although these interaction effects indicate that changes over time varied as a function of feedback condition, they do not specify the source or direction of these differences. Post-hoc comparisons are reported only for outcome variables that demonstrated statistically significant main or interaction effects. For variables where omnibus tests were not significant, post-hoc comparisons are provided descriptively and should be interpreted with caution.

Bonferroni-adjusted pairwise comparisons were performed on pre-to-post gain scores for SRL strategies that showed significant interaction effects. These analyses compared the generative AI feedback group (GenAI-F), the automated feedback group (AF), and the instructor-mediated feedback group (IMF). This approach enabled a precise examination of whether observed gains could be attributed to specific feedback conditions rather than to general practice or time effects.

The Bonferroni correction was applied to control for inflated Type I error resulting from multiple pairwise comparisons. Given the multidimensional nature of SRL and the number of strategies examined, a conservative adjustment was necessary to enhance the reliability and replicability of the findings. The Bonferroni procedure was selected due to its transparency and common use in experimental educational research [32].

In addition to adjusted significance levels, effect sizes were reported to indicate the practical magnitude of group differences. Reporting both adjusted p values and effect sizes supports a more meaningful interpretation of educational impact, which is particularly important in sustainability-oriented research that emphasizes durable and transferable learning outcomes as shown in Table 1.

**Table 1:** Bonferroni-adjusted pairwise comparisons of SRL strategy gain scores.

SRL Strategy	Group Comparison	Mean Difference (Δ)	SE	p (adj.)	Cohen’s d
Idea planning	GenAI-F – AF	0.48	0.14	.004	0.71
	GenAI-F – IMF	0.76	0.16	<.001	1.02
	AF – IMF	0.28	0.15	.184	0.39
Goal-oriented monitoring & evaluation	GenAI-F – AF	0.42	0.13	.007	0.65
	GenAI – IMF	0.69	0.15	<.001	0.94
	AF – IMF	0.27	0.14	.163	0.38
Interest enhancement	GenAI-F – AF	0.36	0.12	.011	0.59
	GenAI-F – IMF	0.58	0.14	.002	0.82
	AF – IMF	0.22	0.13	.241	0.33

Note. Δ represents the mean pre-test to post-test gain score. Reported p values are Bonferroni-adjusted.

Bonferroni-adjusted post-hoc results indicated that learners in the GenAI-F condition achieved significantly greater gains in idea planning, goal-oriented monitoring and evaluation, and interest enhancement than learners in both the AF and IMF conditions. No statistically significant differences were observed between the AF and IMF groups. Effect sizes ranged from medium to large, indicating a meaningful advantage of generative AI feedback in fostering metacognitive and motivational dimensions of SRL.

Prior to the intervention, descriptive statistics showed that the three groups were comparable in their reported use of SRL strategies and overall writing performance. Pre-test mean scores for overall writing were similar across the IMF group (M = 75.18, SD = 5.62), the AF group (M = 75.41, SD = 4.38), and the GenAI-F group (M = 75.66, SD = 3.95). Comparable patterns were also observed across the five writing subcomponents: content, organization, vocabulary, language use, and mechanics.

To confirm group equivalence statistically, one-way ANOVAs were conducted on the nine SRL strategy dimensions, overall writing scores, and writing sub-scores. No significant differences were found among the three groups on any pre-test measure (p > .05). These findings indicate that participants entered the study with comparable levels of SRL strategy use and writing proficiency, providing a robust foundation for interpreting the effects of feedback condition in subsequent analyses.

### Effects of feedback type on self-regulated learning (RQ1).

To address the first research question, which examines changes in self-regulated learning strategies over time across feedback conditions, mixed factorial analyses of variance were conducted for the nine dimensions of self-regulated learning (SRL) strategies. Time (pre-test vs. post-test) was treated as a within-subject factor, and feedback type (instructor-mediated feedback [IMF], automated feedback [AF], and generative AI feedback [GenAI-F] was included as a between-subject factor.

As shown in Table 2, statistically significant main effects of time were observed for several cognitive and metacognitive strategies, including text processing, knowledge rehearsal, idea planning, goal-oriented monitoring and evaluation, and feedback handling (p < .01). The associated partial eta squared values ranged from.17 to.53, indicating medium to large effects. These findings suggest that sustained engagement in academic writing tasks over the semester supported overall growth in SRL strategy use across all instructional conditions.

**Table 2:** Results of mixed factorial ANOVAs on self-regulated learning strategies.

Dimension	Strategy	Effect	F	p	Partial η²
**Cognition**	Text processing	Time	36.84	<.001	0.31
		Feedback type	0.91	.405	0.02
		Time × Feedback	1.12	.331	0.03
	Knowledge rehearsal	Time	34.29	<.001	0.30
		Feedback type	0.58	.562	0.01
		Time × Feedback	0.49	.616	0.01
**Metacognition**	Idea planning	Time	91.76	<.001	0.53
		Feedback type	1.07	.346	0.03
		Time × Feedback	4.62	.013	0.11
	Goal-oriented monitoring & evaluation	Time	21.43	<.001	0.21
		Feedback type	0.19	.829	0.01
		Time × Feedback	5.18	.008	0.13
**Social behavior**	Peer learning	Time	9.84	.003	0.11
		Feedback type	0.27	.764	0.01
		Time × Feedback	0.61	.545	0.02
	Feedback handling	Time	15.67	<.001	0.17
		Feedback type	0.12	.887	0.00
		Time × Feedback	1.03	.361	0.03
**Motivational regulation**	Interest enhancement	Time	6.02	.016	0.08
		Feedback type	1.34	.268	0.04
		Time × Feedback	4.97	.009	0.12
	Motivational self-talk	Time	3.11	.081	0.05
		Feedback type	0.41	.663	0.01
		Time × Feedback	0.44	.645	0.01
	Emotional control	Time	4.09	.046	0.06
		Feedback type	0.73	.486	0.02
		Time × Feedback	0.39	.679	0.01

Beyond these general developmental trends, significant Time by Feedback Type interaction effects were identified for goal-oriented monitoring and evaluation and for interest enhancement. The interaction effect for goal-oriented monitoring and evaluation explained approximately 13 percent of the variance, indicating that changes in learners’ metacognitive regulation differed as a function of feedback condition. A comparable interaction effect was observed for interest enhancement, accounting for about 12 percent of the variance. This pattern suggests that learners’ motivational engagement with writing tasks evolved differently depending on the type of feedback they received.

In contrast, no significant interaction effects were found for cognitive strategies such as text processing and knowledge rehearsal, nor for social-behavioral strategies such as peer learning and feedback handling. Similarly, motivational self-talk and emotional control did not show differential change across feedback conditions. Taken together, these results indicate that while engagement in writing instruction generally promoted SRL development, generative AI feedback exerted a distinctive influence on specific metacognitive and motivational regulation strategies that are central to sustained and autonomous learning.

To further interpret the significant Time by Feedback Type interaction effects, Bonferroni-adjusted simple effects analyses were conducted on post-test SRL gain scores. These analyses aimed to clarify the direction and magnitude of between-group differences in strategies that exhibited differential developmental trajectories over time.

As reported in Table 3, statistically significant between-group differences emerged for goal-oriented monitoring and evaluation and for interest enhancement. Learners in the GenAI-F condition demonstrated significantly greater gains in interest enhancement than those receiving AF feedback, indicating stronger motivational engagement when feedback was dialogic and explanatory. In contrast, learners in the IMF condition showed significantly greater improvement in goal-oriented monitoring and evaluation than those in the AF group. This pattern may reflect Turkish L2 learners’ increased reliance on self-monitoring and evaluative strategies in instructional contexts where automated feedback is absent and accountability for performance is internalized, particularly within exam-oriented higher education settings.

**Table 3:** Between-group simple effects analyses of SRL strategy gains at post-test.

SRL Strategy	F(2, 81)	p	Partial η²	Significant Pairwise Comparisons (Bonferroni-adjusted)
**Text processing**	0.66	.522	.02	n.s.
**Knowledge rehearsal**	0.24	.784	.01	n.s.
**Idea planning**	0.16	.856	.01	n.s.
**Goal-oriented monitoring and evaluation**	3.75	.028	.09	IMF > AF (MD = 0.76, p = .048)
**Peer learning**	0.59	.557	.01	n.s.
**Feedback handling**	0.17	.841	.01	n.s.
**Interest enhancement**	4.01	.022	.09	GenAI-F > AF (MD = 0.88, p = .029)
**Motivational self-talk**	0.36	.698	.01	n.s.
**Emotional control**	0.45	.640	.01	n.s.

Note. Bonferroni-adjusted p values are reported. n.s. = not significant.

No statistically significant group differences were observed for the remaining SRL strategies, suggesting that the differential impact of feedback type was selective rather than uniform across SRL dimensions.

Within-group comparisons further clarified the developmental patterns associated with each feedback condition. Paired-samples t-tests were conducted to examine pre-test to post-test changes in SRL strategies for each group, as presented in Table 4.

**Table 4:** Within-group simple effects analyses of SRL strategy development.

Group	SRL Strategy	MD (Pre–Post)	SD	t	p	Cohen’s d
**IMF**	Text Processing	−0.54	0.70	−3.51	.002	0.77
	Knowledge Rehearsal	−0.48	0.68	−3.51	.002	0.79
	Idea Planning	−0.96	0.83	−5.82	<.001	1.20
	Goal-Oriented Monitoring & Evaluation	−0.81	0.70	−4.19	<.001	1.15
	Peer Learning	−0.45	0.75	−3.01	.006	0.50
	Feedback Handling	+0.41	0.69	3.13	.004	0.59
	Interest Enhancement	−0.27	0.68	−2.00	.056	0.41
	Motivational Self-talk	−0.08	0.64	−0.58	.567	0.13
	Emotional Control	−0.12	0.62	−0.74	.468	0.19
**AF**	Text Processing	−0.58	0.76	−2.85	.008	0.53
	Knowledge Rehearsal	−0.72	0.80	−3.78	.001	0.79
	Idea Planning	−0.84	0.82	−4.10	<.001	1.00
	Goal-Oriented Monitoring & Evaluation	−0.29	0.77	−1.62	.117	0.29
	Peer Learning	−0.21	0.73	−1.06	.300	0.22
	Feedback Handling	+0.33	0.76	1.71	.099	0.33
	Interest Enhancement	−0.24	0.69	−1.18	.247	0.21
	Motivational Self-talk	−0.09	0.61	−0.52	.610	0.09
	Emotional Control	−0.08	0.66	0.51	.611	0.14
**GenAI-F**	Text Processing	−0.75	0.79	−4.44	<.001	0.76
	Knowledge Rehearsal	−0.84	0.83	−3.25	.003	0.64
	Idea Planning	−1.27	0.84	−7.13	<.001	1.78
	Goal-Oriented Monitoring & Evaluation	−0.32	0.79	−1.32	.198	0.28
	Peer Learning	−0.41	0.85	−1.87	.072	0.44
	Feedback Handling	+0.48	0.82	2.11	.044	0.45
	Interest Enhancement	−0.66	0.78	−2.46	.020	0.48
	Motivational Self-talk	−0.29	0.80	−1.72	.097	0.36
	Emotional Control	+0.51	0.78	2.11	.044	0.52

Students in the AF group demonstrated significant gains in text processing, knowledge rehearsal, and idea planning, with medium to large effect sizes. However, no significant changes were observed in motivational regulation strategies, suggesting that form-focused automated feedback primarily supported lower-level cognitive regulation rather than motivational engagement.

The GenAI-F group exhibited significant gains in text processing, knowledge rehearsal, and idea planning, with the largest effect observed for idea planning. This finding highlights the strong planning-oriented affordances of generative AI feedback. In addition, students in this group reported a significant increase in interest enhancement, indicating improved motivational engagement with writing tasks. At the same time, small but significant declines were observed in feedback handling and emotional control. These changes may reflect increased reliance on AI-generated guidance or heightened cognitive and affective demands associated with interacting with generative systems.

The IMF group showed moderate to large improvements in several cognitive and metacognitive strategies, including idea planning and goal-oriented monitoring and evaluation. However, a significant decline in feedback handling was also observed, underscoring the constraints faced by learners when opportunities for individualized feedback are limited.

Taken together, these within-group findings indicate that GenAI-F exerted the strongest influence on planning-related and motivational SRL strategies, whereas AF feedback primarily supported surface-level cognitive regulation. Instructor-mediated feedback promoted metacognitive monitoring but offered more limited support for sustained motivational engagement.

### Effects of feedback type on writing performance (RQ2).

To address Research Question 2, which focuses on changes in writing performance over time, a series of 2 × 3 mixed factorial analyses of variance were conducted, with time (pre-test vs. post-test) as the within-subject factor and feedback type (IMF, AF, GenAI-F) as the between-subject factor. Analyses were performed for overall writing scores and for five writing subcomponents, as summarized in Table 5.

**Table 5:** Mixed factorial ANOVA results for overall writing performance and subcomponents.

Writing Outcome	Effect	F(2, 81)	p	Partial η²
**Overall score**	Time	20.65	<.001	.20
	Feedback type	1.93	.152	.05
	Time × Feedback	6.00	.004	.13
**Content**	Time	21.23	<.001	.21
	Feedback type	3.18	.047	.07
	Time × Feedback	5.10	.008	.11
**Organization**	Time	2.10	.152	.03
	Feedback type	1.64	.200	.04
	Time × Feedback	2.48	.090	.06
**Vocabulary**	Time	41.33	<.001	.34
	Feedback type	1.36	.264	.03
	Time × Feedback	1.79	.174	.04
**Language use**	Time	1.19	.279	.01
	Feedback type	1.06	.351	.03
	Time × Feedback	2.05	.136	.05
**Mechanics**	Time	1.18	.314	.03
	Feedback type	2.72	.072	.06
	Time × Feedback	2.72	.072	.06

The analyses revealed significant main effects of time on overall writing performance as well as on content, vocabulary, and mechanics (p < .001), with medium to large effect sizes. These findings indicate that students’ writing performance improved over the semester across all instructional conditions, reflecting cumulative learning effects associated with sustained writing practice.

A significant main effect of feedback type was observed for content scores, suggesting that different feedback modalities had differential impacts on idea development and elaboration. More importantly, statistically significant Time by Feedback Type interaction effects emerged for overall writing scores and content. These interaction effects accounted for 13 percent and 11 percent of the variance, respectively, indicating that the magnitude of writing improvement differed meaningfully across feedback conditions.

Follow-up simple effects analyses demonstrated that, at the post-test, learners in the GenAI-F condition outperformed those in the IMF condition on overall writing scores and mechanics. In addition, the GenAI-F group achieved significantly higher overall and content scores than the AF group. These findings point to the added value of generative AI feedback in supporting higher-order writing processes, particularly content development and argument elaboration, which are areas of persistent difficulty for Turkish L2 learners due to limited opportunities for extended and iterative writing.

Within-group analyses further revealed differentiated patterns of development. The AF group demonstrated significant gains in content and mechanics, with particularly strong effects for content, indicating the effectiveness of automated feedback for improving surface-level accuracy and clarity. The GenAI-F group showed significant improvements in overall writing performance as well as in content, vocabulary, and mechanics, with consistently large effect sizes across components. In contrast, the IMF group exhibited more modest gains, primarily in content and language-related sub-scores, consistent with general instructional effects observed in teacher-led writing contexts.

Overall, these findings suggest that while all feedback conditions contributed to writing development over time, generative AI feedback was associated with broader and more substantial gains across multiple dimensions of writing performance, highlighting its potential role in supporting sustainable and scalable writing instruction in higher education.

### Moderating role of proficiency (RQ3).

Unlike RQ1 and RQ2, which examine changes over time, RQ3 focuses on post-test outcomes to assess the moderating role of proficiency. To address Research Question 3, a two-way analysis of variance was conducted on overall writing gain scores. The results revealed a statistically significant main effect of feedback type, as well as a significant interaction between feedback type and L2 proficiency level. These findings indicate that the effectiveness of feedback varied depending on learners’ proficiency.

Simple effects analyses showed that within the AF condition, advanced-proficiency learners achieved significantly greater writing gains than intermediate-proficiency learners. This pattern suggests that learners with higher linguistic competence were better able to interpret and apply form-focused automated feedback. In contrast, within the GenAI-F condition, intermediate-proficiency learners demonstrated significantly greater writing gains than their counterparts in the AF condition. This finding highlights the scaffolding role of generative AI feedback, which appears particularly beneficial for Turkish L2 learners who possess foundational language knowledge but require support with idea generation, organization, and elaboration.

Taken together, these results indicate that GenAI-F is especially effective for intermediate-level learners, whereas AF feedback appears more suitable for advanced learners. This proficiency-sensitive pattern underscores the importance of aligning feedback technologies with learner characteristics in sustainable and inclusive L2 writing instruction.

To further clarify the nature of the significant interaction effects identified in the mixed factorial analyses, Bonferroni-adjusted post-hoc comparisons were conducted on post-test writing scores across the three feedback conditions. These analyses aimed to identify specific group differences in overall writing performance and its analytic subcomponents. The results are presented in Table 6.

**Table 6:** Bonferroni-adjusted post-test comparisons of writing performance across feedback conditions.

Writing Scores	Group Comparison	Mean Difference (MD)	p
**Overall Score**	IMF vs. AF	1.24	.214
	IMF vs. GenAI-F	3.15	.008*
	GenAI-F vs. AF	3.72	.014*
**Content**	IMF vs. AF	0.42	.397
	IMF vs. GenAI-F	1.09	.006**
	GenAI-F vs. AF	0.97	.009**
**Organization**	IMF vs. AF	0.31	.614
	IMF vs. GenAI-F	0.54	.338
	GenAI-F vs. AF	0.23	.721
**Vocabulary**	IMF vs. AF	0.46	.281
	IMF vs. GenAI-F	0.79	.094
	GenAI-F vs. AF	0.33	.447
**Language Use**	IMF vs. AF	0.29	.512
	IMF vs. GenAI-F	0.48	.236
	GenAI-F vs. AF	0.19	.689
**Mechanics**	IMF vs. AF	0.72	.041*
	IMF vs. GenAI-F	0.98	.018*
	GenAI-F vs. AF	0.26	.604

p < .05, ** p < .01.

The post-hoc results indicate that learners in the GenAI-F condition outperformed those in the IMF condition on overall writing performance and content. In addition, GenAI-F learners achieved significantly higher overall and content scores than those in the AF condition. For mechanics, both AF and GenAI-F groups demonstrated advantages over the IMF group, suggesting that technology-mediated feedback supported surface- level accuracy more effectively than instructor-mediated feedback alone.

To examine how writing performance developed over time within each instructional condition, paired-samples t-tests were conducted for overall writing scores and the five analytic subcomponents. The results are presented in Table 7.

**Table 7:** Within-group comparisons of writing performance from pre-test to post-test.

Group	Writing Dimension	Mean Difference (Post–Pre)	t	p	Cohen’s d
**IMF**	Overall	1.88	2.41	.021*	0.45
	Content	1.02	3.19	.003**	0.71
	Organization	0.38	1.21	.236	0.24
	Vocabulary	0.56	2.02	.049*	0.40
	Language Use	0.49	1.98	.054	0.39
	Mechanics	0.44	2.17	.035*	0.43
**AF**	Overall	2.76	3.84	<.001***	0.71
	Content	1.45	5.62	<.001***	1.29
	Organization	0.51	1.87	.072	0.35
	Vocabulary	0.63	2.14	.041*	0.40
	Language Use	0.57	2.01	.053	0.38
	Mechanics	0.91	3.26	.003**	0.60
**GenAI-F**	Overall	5.48	7.92	<.001***	1.48
	Content	2.84	11.63	<.001***	3.00
	Organization	0.88	2.95	.006**	0.55
	Vocabulary	1.26	4.78	<.001***	0.95
	Language Use	0.74	2.41	.022*	0.45
	Mechanics	1.05	3.88	<.001***	0.72

p < .05, p < .01, p < .001.

Viewed through the lens of sustainability and lifelong learning, these findings highlight feedback-mediated self-regulated learning as a central mechanism for developing durable writing competence beyond short-term performance gains. Learners in the GenAI-F condition, who demonstrated marked growth in metacognitive regulation, particularly idea planning and goal-oriented monitoring, alongside increased motivational engagement, also achieved the most substantial and transferable improvements in writing quality, especially in content development.

These gains suggest that generative AI feedback can support learners in developing the capacity to independently plan, evaluate, and refine their writing over time. Such competencies are essential for sustainable language learning in digitally mediated higher education contexts. In contrast, improvements in the AF condition were largely confined to mechanics and form-focused accuracy, reflecting gains in procedural efficiency rather than broader self-regulatory control. While these outcomes remain pedagogically valuable, their limited impact on higher-order SRL dimensions may restrict learners’ long-term adaptability as autonomous writers.

The comparatively modest progress observed in the IMF condition further underscores the importance of feedback practices that actively foster learners’ self-regulation, motivation, and agency. Collectively, the results suggest that AI-empowered feedback systems designed to cultivate SRL strategies can contribute to more sustainable models of L2 writing instruction by equipping learners with self-directed skills essential for continuous learning across academic, professional, and lifelong contexts.

To further examine whether learners’ L2 proficiency moderated the effectiveness of different feedback types, a two-way analysis of variance was conducted with feedback type (instructor-mediated feedback, automated feedback, and generative AI feedback) and CEFR-based proficiency level (intermediate vs. advanced) as between-subject factors, and overall writing gain scores as the dependent variable. Figure 1 (S1 Fig) illustrates the interaction pattern by comparing post–pre writing gains across feedback conditions for learners at different CEFR levels.

As illustrated in Fig 1, a clear interaction pattern emerged between feedback type and CEFR proficiency level. For advanced learners (CEFR C1), both AI-supported conditions led to greater writing gains than the no-feedback condition, with AF feedback yielding particularly strong improvements. In contrast, intermediate learners (CEFR B1–B2) benefited most from GenAI-F, showing substantially larger gains than their counterparts in the AF and IMF groups. This divergence suggests that GenAI-F may provide more adaptive scaffolding for learners with developing proficiency, while advanced learners are better positioned to capitalize on form-focused AF feedback. Overall, the figure visually substantiates the statistically significant moderation effect reported in the ANOVA results and highlights the importance of aligning AI feedback types with learners’ proficiency levels to support equitable and sustainable writing development in higher education. From a sustainability perspective, these findings suggest that GenAI-F can redistribute cognitive responsibility to learners, fostering durable self-regulation skills rather than short-term performance gains.

## Discussion

This study examined how different forms of AI-mediated feedback influenced Turkish L2 learners’ self-regulated learning (SRL) strategies and writing development over time, with particular attention to sustainability and learner equity. Across all feedback conditions, significant gains were observed in multiple SRL dimensions, confirming prior research demonstrating that sustained academic writing practice fosters strategic engagement and learner responsibility [16,33]. More importantly, significant interaction effects between time and feedback type emerged for specific SRL dimensions, indicating that feedback modality plays a meaningful role in shaping learners’ strategic development. In interpreting these findings, only statistically significant effects are treated as evidence of differential impact, while non-significant differences are discussed cautiously as descriptive trends.

The interaction effects observed for metacognitive regulation were primarily driven by gains in the idea planning and goal-oriented monitoring subscales. The effect sizes for these dimensions were in the moderate range, suggesting that the improvements were not only statistically significant but also pedagogically meaningful. Learners in the GenAI-F condition demonstrated a greater capacity to generate ideas, articulate writing goals, and align revisions with rhetorical intent over time. These findings are consistent with socio-cognitive models of self-regulated learning, which emphasize explanatory, dialogic, and goal-referenced feedback as key mechanisms supporting metacognitive regulation [34,35]. Unlike automated feedback systems that primarily target surface-level linguistic accuracy, generative AI feedback provided adaptive explanations and revision-oriented prompts that encouraged learners to reflect on content development and planning decisions, thereby functioning as an external metacognitive scaffold [8,11].

With respect to motivational regulation, the significant interaction effect for interest enhancement was accompanied by a small to moderate effect size. Although smaller in magnitude than the metacognitive gains, this effect is educationally consequential given the cumulative nature of motivation in sustained writing practice. The dialogic and responsive nature of GenAI-F appears to have increased perceived task value and learner engagement, supporting learners’ willingness to invest effort across successive writing cycles. This finding aligns with previous research indicating that feedback which enhances learner autonomy and perceived control contributes to motivational sustainability and long-term engagement [19,20]. In the Turkish higher education context, where writing instruction is often exam-driven and instructor-centered, such motivational regulation is particularly important for sustaining learner autonomy.

In contrast, no significant interaction effects were found for the cognitive regulation or social regulation subscales, and the associated effect sizes were negligible. This pattern suggests that these dimensions may be less sensitive to feedback modality alone or may require explicit instructional scaffolding and longer timeframes to develop. Previous research has similarly shown that cognitive and social SRL strategies are strongly influenced by task design and opportunities for peer interaction rather than feedback alone [12,29]. Given the predominantly individual and product-oriented nature of academic writing tasks in Turkish universities, opportunities for peer-supported regulation remain limited, which likely constrained the development of social SRL strategies regardless of feedback type.

Writing performance results further reinforced the sustainability value of generative AI feedback. Learners receiving GenAI-F demonstrated the largest overall gains in writing quality, particularly in content development, which showed moderate to large effect sizes. Content development is widely regarded as a key indicator of transferable academic literacy and higher-order writing competence [36]. In contrast, automated feedback primarily supported mechanics and surface-level accuracy, yielding small to moderate effects in these subcomponents. This pattern aligns with previous findings indicating that automated feedback systems are effective for form-focused revision but offer limited support for meaning-level writing development [3,4]. Together, these results highlight the complementary yet distinct pedagogical roles of different AI feedback tools.

The moderation effect of language proficiency provides further insight into equitable AI deployment. Higher-proficiency learners benefited more from automated feedback, likely due to stronger linguistic resources and greater capacity to interpret decontextualized corrective input. In contrast, intermediate-level learners demonstrated greater gains under GenAI-F conditions, suggesting that adaptive explanations and dialogic interaction may mitigate proficiency-related barriers to feedback uptake. This finding is consistent with research showing that learners’ engagement with feedback is mediated by linguistic proficiency and metacognitive resources [9,13]. From an equity perspective, generative AI feedback may therefore reduce proficiency-based disparities by offering differentiated support aligned with learners’ needs.

From a sustainability standpoint, the findings suggest that GenAI-F can function as a scalable metacognitive and motivational scaffold that supports strategic development without increasing instructor workload. However, the limited impact of automated feedback on higher-order SRL highlights the importance of embedding AI tools within pedagogical frameworks that explicitly promote reflection, self-evaluation, and learner agency [27]. Sustainable AI integration thus depends not only on technological access but also on instructional design and feedback literacy. While the findings highlight the pedagogical sustainability of generative AI feedback in fostering self-regulated learning, the implications for instructor workload require more cautious interpretation. Although AI-mediated feedback offers scalable support for iterative writing practice, the present study did not directly measure instructional time, feedback provision effort, or workload redistribution across conditions. Nevertheless, from a theoretical and practical perspective, generative AI systems have the potential to partially offload routine feedback functions, particularly those related to initial drafting, idea generation, and surface-level revision. This may allow instructors to reallocate their time toward higher-value pedagogical activities, such as providing individualized strategic guidance, facilitating peer interaction, and supporting metacognitive development. In large-scale higher education contexts such as Türkiye, where heavy teaching loads constrain opportunities for formative feedback, such redistribution may represent a critical dimension of sustainable pedagogy. Future research should therefore adopt mixed-method or time-tracking designs to examine how AI integration affects instructor workload, feedback practices, and pedagogical decision-making in real classroom settings.

Overall, the findings indicate that proficiency-sensitive and pedagogically grounded AI-mediated feedback can support sustainable, equitable, and lifelong-oriented writing instruction in Turkish higher education. By demonstrating differential effects across SRL subscales and writing dimensions, this study contributes empirical evidence to ongoing debates on how AI technologies can be integrated responsibly and effectively into L2 writing pedagogy.

A limitation of the study concerns the use of ChatGPT-4 for the initial scoring of writing tasks. Although rubric-based prompting and human validation procedures were employed to enhance consistency and reliability, ChatGPT is not a fully validated assessment instrument. In addition, its dual role as both a feedback provider in the intervention phase and a scoring assistant introduces a potential source of methodological bias. While steps were taken to standardize scoring and reduce this risk, future research should incorporate fully independent human rating procedures or validated automated scoring systems to strengthen assessment validity.

Another limitation concerns treatment fidelity and learner engagement with the feedback tools. Although structured training, in-class monitoring, and post-task checks were used to promote consistent implementation, the study did not include detailed usage analytics (e.g., interaction logs, frequency of revisions, or time-on-task). As a result, it is not possible to determine the extent to which individual differences in engagement with Grammarly or ChatGPT influenced the observed outcomes. Future research should incorporate process-tracing methods, such as system logs, screen recordings, or learning analytics, to provide a more precise account of how learners interact with AI-mediated feedback.

A further methodological consideration concerns the use of multiple univariate analyses rather than a multivariate approach. While separate ANOVAs allowed for detailed examination of individual SRL dimensions and writing components, this approach may increase the risk of Type I error. Although Bonferroni corrections were applied to mitigate this risk, future research could employ MANOVA or MANCOVA to account for interdependencies among outcome variables and provide a more conservative test of group differences.

A methodological distinction should be noted between the analyses addressing development over time (RQ1 and RQ2) and the moderation analysis (RQ3), which was based on post-test outcomes. While this approach allows for a clear examination of proficiency effects on final performance, future research may consider longitudinal moderation models to capture how proficiency influences developmental trajectories.

## Conclusion

Grounded in sociocognitive theory and sustainability-oriented higher education policy, this study investigated the effects of automated feedback and generative AI feedback on Turkish L2 learners’ self-regulated learning strategies and writing performance, while examining the moderating role of CEFR-aligned proficiency levels.

The findings demonstrate that AI-empowered feedback can foster sustainable writing development when it strengthens learners’ self-regulatory capacities rather than focusing solely on surface-level correction. While all groups benefited from instruction over time, GenAI-F showed distinctive advantages in promoting metacognitive planning and motivational engagement, which were associated with stronger and more durable improvements in writing quality, particularly in content development.

Importantly, the effectiveness of AI feedback was proficiency-dependent. Intermediate learners benefited most from GenAI-F due to its adaptive scaffolding and explanatory affordances, whereas advanced learners showed greater gains under automated feedback. This pattern highlights the importance of equitable, learner-sensitive AI integration in mass higher education systems.

From a sustainability perspective, AI-supported writing pedagogy offers potential to reduce instructor workload, promote learner autonomy, and support differentiated instruction at scale. However, the findings also underscore the necessity of pedagogically guided AI use to ensure that social interaction, reflection, and self-evaluation remain central to writing instruction.

Within the context of Türkiye’s digital transformation agenda in higher education, this study provides evidence that AI tools should be strategically embedded within curricula rather than adopted as stand-alone solutions. Sustainable L2 writing instruction emerges from the balanced alignment of technology, pedagogy, and learner agency. However, claims regarding the capacity of AI-supported feedback to reduce instructor workload should be interpreted with caution, as this study did not include direct measures of instructional effort or time allocation. Future research should explicitly investigate how AI-mediated feedback reshapes teacher roles, workload distribution, and instructional efficiency, thereby providing a more comprehensive understanding of sustainability at both learner and institutional levels.

Despite limitations related to intervention duration and reliance on self-report measures, the study offers robust evidence that GenAI-F, when proficiency-aligned and pedagogically framed, can serve as a catalyst for sustainable L2 writing development. By foregrounding self-regulated and lifelong learning capacities, AI-supported feedback can meaningfully contribute to the broader sustainability mission of higher education in Türkiye and comparable contexts.

The findings of this study offer several pedagogically significant implications for sustainable L2 writing instruction in Turkish higher education, particularly in relation to Türkiye’s digital transformation agenda, YÖK’s strategic priorities [37]., and universities’ responsibility to foster lifelong, self-regulated learners. In this context, sustainability extends beyond environmental considerations to include pedagogical durability, learner autonomy, and equitable access to high-quality feedback in mass higher education.

First, the differential effects of GenAI-F on metacognitive and motivational SRL strategies highlight its potential to address a persistent structural challenge in Turkish universities: large class sizes and limited opportunities for individualized formative feedback in compulsory English courses. As noted in YÖK policy reports [38,39], heavy teaching loads often constrain instructors’ capacity to provide sustained feedback on student writing. The present findings suggest that GenAI-F can function as a scalable supplementary feedback mechanism, supporting idea planning, goal setting, and interest regulation without increasing instructor workload. From a sustainability perspective, this aligns with resource-efficient pedagogy, where digital tools are leveraged to maintain instructional quality over time.

Second, the observed relationship between SRL strategy development and writing performance underscores the need to shift Turkish L2 writing pedagogy from predominantly product-oriented assessment toward process-oriented and self-regulatory approaches. Writing instruction in many Turkish universities continues to emphasize grammatical accuracy and exam-driven outcomes, shaped by centralized assessment traditions and CEFR alignment [38]. The present results indicate that when learners are supported in regulating planning, monitoring, and motivation particularly through dialogic GenAI-F, writing development becomes more durable and transferable across tasks. Embedding SRL-focused pedagogy into university English curricula may therefore better support sustainable academic literacy development than short-term performance gains.

Third, the moderating role of CEFR proficiency level has important implications for equity and inclusivity in Türkiye’s digital education initiatives. Intermediate-level learners (CEFR B1–B2), who constitute the majority of first-year undergraduates in state universities, benefited more from GenAI-F than from AF feedback, whereas advanced learners (C1) showed greater gains under AF conditions. This finding cautions against one-size-fits-all approaches to AI integration, which may inadvertently amplify achievement gaps. Sustainable implementation requires differentiated feedback models, in which GenAI tools scaffold learners with developing proficiency, while more advanced learners are encouraged to critically engage with form-focused automated feedback.

Fourth, the mixed effects observed in social and emotional regulation strategies highlight the need for pedagogically guided AI use. While GenAI-F enhanced interest regulation, it was also associated with reduced reliance on peer- and instructor-mediated feedback. In the Turkish context, where collaborative learning and instructor guidance remain culturally valued, sustainable AI integration should be framed as complementary rather than substitutive. Combining GenAI-F with structured peer review, reflective writing tasks, and explicit SRL strategy instruction may help preserve the social dimension of learning while capitalizing on AI affordances.

Finally, these findings align closely with Türkiye’s broader sustainability and lifelong learning objectives articulated in national higher education and digitalization policies [39,40]. By fostering learners’ capacity to self-regulate their writing through informed and reflective use of AI feedback, universities can support the development of adaptive, autonomous, and digitally literate graduates prepared for continuous learning beyond formal education. Sustainable L2 writing pedagogy in Türkiye, therefore, is not merely a matter of technological adoption, but of cultivating enduring learner capacities that remain functional across evolving academic, professional, and technological contexts.

In sum, this study suggests that strategically differentiated, SRL-oriented integration of GenAI-F holds substantial promise for advancing sustainable L2 writing pedagogy in Turkish higher education. Future institutional efforts should focus not only on technology deployment, but also on pedagogical alignment, instructor professional development, and policy-level guidance to ensure that AI-enhanced writing instruction contributes meaningfully to long-term educational sustainability.

## Supporting information

S1 Fig Moderating effect of CEFR proficiency level on overall writing gains across feedback conditions. (PNG)

S1 File Anonymized participant dataset. Dataset containing participant-level data used in the analyses, including writing performance scores, writing subcomponent scores, group assignments, proficiency classifications, and self-regulated learning questionnaire responses.
(XLSX)

S2 File Codebook for study variables. Detailed description of all variables, coding procedures, scale labels, and data structure used in the dataset.
(DOCX)

S3 File Statistical analysis syntax. SPSS syntax file used to conduct the statistical analyses reported in the manuscript.
(DOCX)

S4 File R script. R script used for data preparation, statistical analyses, and generation of figures and supplementary outputs reported in the study.
(DOCX)

S5 File Structural Equation Modeling (SEM) script. SEM analysis script and model specifications used for supplementary structural equation modeling procedures reported in the study.
(DOCX)
