# Supplementary figures and images for: Sustainable L2 writing pedagogy in Turkish higher education: Effects of AI-mediated feedback on self-regulated learning and writing performance
Source: PLoS One. 2026 Jul 14;21(7):e0344618. doi: 10.1371/journal.pone.0344618 (PMC13367666; doi:10.1371/journal.pone.0344618)

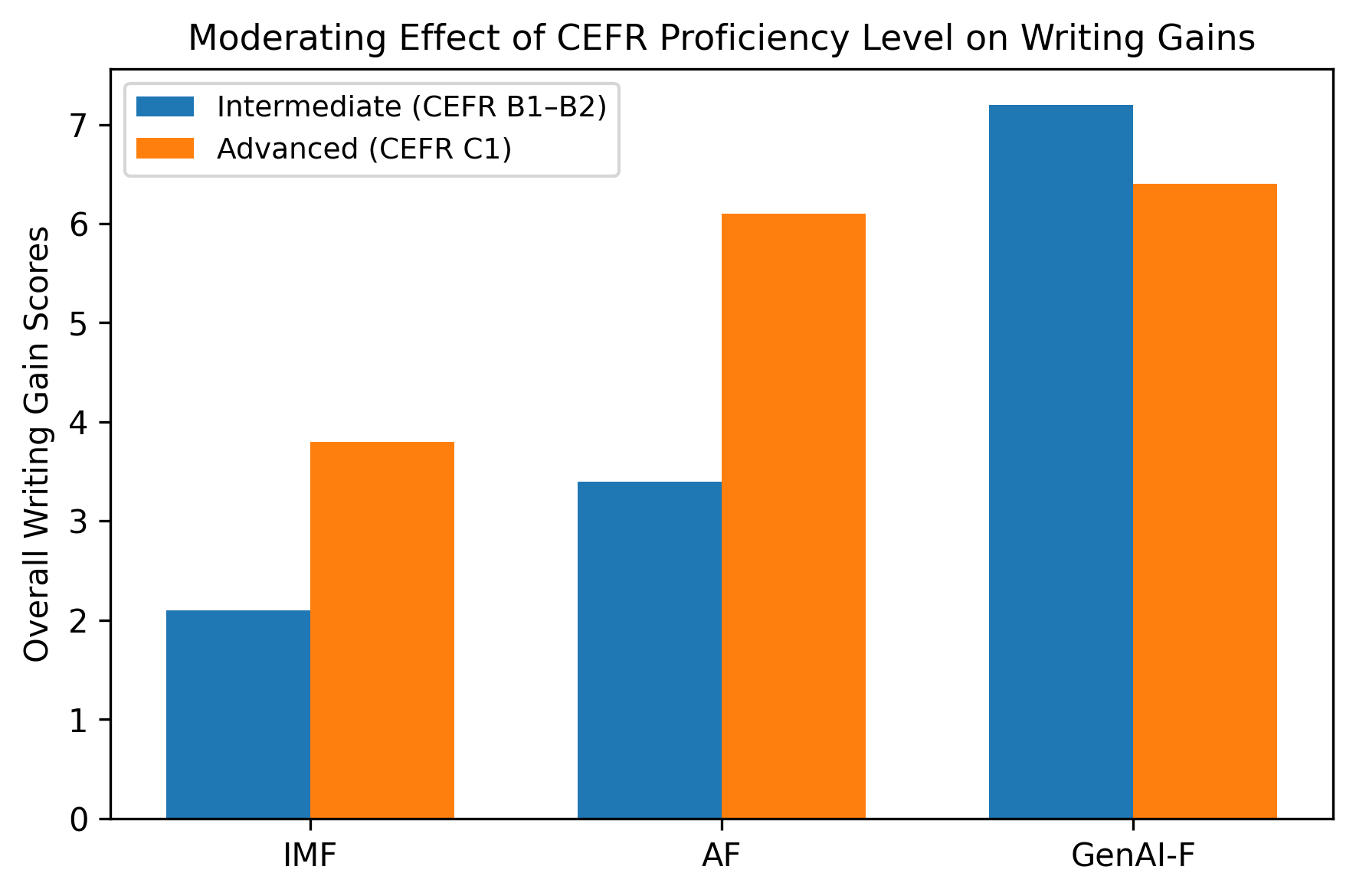

Supplement: S1 Fig — (PNG) [file pone.0344618.s001.png]
