# Supplementary material for: Sustainable L2 writing pedagogy in Turkish higher education: Effects of AI-mediated feedback on self-regulated learning and writing performance
Source: PLoS One. 2026 Jul 14;21(7):e0344618. doi: 10.1371/journal.pone.0344618 (PMC13367666; doi:10.1371/journal.pone.0344618)
Supplement: S2 File — Detailed description of all variables, coding procedures, scale labels, and data structure used in the dataset. (DOCX) [file pone.0344618.s003.docx]

**S2 Codebook**

**Description of Dataset Variables**

**Dataset Title**

Dataset for: The Effects of Feedback Types on Writing Performance and Self-Regulated Learning

**General Description**

This dataset contains anonymized, participant-level data (N = 84) used to examine the effects of different feedback types on writing performance and self-regulated learning (SRL). The dataset includes group assignment, proficiency level, pre-test and post-test writing scores, writing subcomponents, and SRL questionnaire responses.

**Variable Descriptions**

**1. Identification Variables - ID**

- Description: Unique participant identifier
- Type: Numeric
- Range: 1–84

**2. Group Variables - Group**

- Description: Experimental condition
- Type: Categorical (String)
- Values:
  - IMF = Indirect Metalinguistic Feedback
  - AF = Automated Feedback
  - GenAI-F = Generative AI Feedback

**3. Proficiency Variable - Proficiency**

- Description: Participant English proficiency level
- Type: Categorical (String)
- Values:
  - B1-B2 = Intermediate
  - B2-C1 = Upper-intermediate

**4. Writing Performance Variables - Overall Scores**

**Pre_Overall**

- Description: Pre-test writing score (overall)
- Type: Continuous
- Scale: 0–100

**Post_Overall**

- Description: Post-test writing score (overall)
- Type: Continuous
- Scale: 0–100

**Writing Subcomponents**

Each subcomponent is scored on a 0–100 scale.

**Pre_Content / Post_Content**

- Description: Content quality score

**Pre_Organization / Post_Organization**

- Description: Organization and coherence score

**Pre_Vocabulary / Post_Vocabulary**

- Description: Vocabulary usage score

**Pre_LanguageUse / Post_LanguageUse**

- Description: Grammar and language use score

**Pre_Mechanics / Post_Mechanics**

- Description: Mechanics (spelling, punctuation) score

**5. Self-Regulated Learning (SRL) Variables**

**SRL_1 – SRL_35**

- Description: Responses to the Writing Strategies for Self-Regulated Learning Questionnaire (WSSRLQ)
- Type: Ordinal (Likert scale)
- Scale:
  - 1 = Strongly Disagree
  - 2 = Disagree
  - 3 = Slightly Disagree
  - 4 = Neutral
  - 5 = Slightly Agree
  - 6 = Agree
  - 7 = Strongly Agree

**6. Derived Variables (Optional for Analysis)**

These variables are not included in the dataset but can be computed:

**Gain_Overall**

- Formula: Post_Overall − Pre_Overall

**Gain_Subcomponents**

- Formula: Post score − Pre score for each subcomponent

**7. Missing Data**

- No missing data are present in this dataset
- All values are complete for statistical analysis

**8. Data Anonymization**

- No personally identifiable information is included
- All data are anonymized in accordance with ethical research standards

**9. Notes for Replication**

- All statistical analyses (ANOVA, post-hoc tests, moderation) can be reproduced using this dataset
- Variables correspond directly to those reported in the manuscript tables and figures
