# Supplementary material for: Sustainable L2 writing pedagogy in Turkish higher education: Effects of AI-mediated feedback on self-regulated learning and writing performance
Source: PLoS One. 2026 Jul 14;21(7):e0344618. doi: 10.1371/journal.pone.0344618 (PMC13367666; doi:10.1371/journal.pone.0344618)
Supplement: S3 File — SPSS syntax file used to conduct the statistical analyses reported in the manuscript. (DOCX) [file pone.0344618.s004.docx]

**S3 File. SPSS Syntax for Statistical Analyses**

**1. Data Import**

GET DATA
/TYPE=XLSX
/FILE='PLoS_dataset_simulated.xlsx'
/SHEET=name 'Sheet1'
/READNAMES=on.

EXECUTE.

**2. Descriptive Statistics**

DESCRIPTIVES VARIABLES=Pre_Overall Post_Overall
/STATISTICS=MEAN STDDEV MIN MAX.æ

**3. Compute Gain Scores**

COMPUTE Gain_Overall = Post_Overall - Pre_Overall.
EXECUTE.

**4. Mixed ANOVA (Group × Time)**

GLM Pre_Overall Post_Overall BY Group
/WSFACTOR=Time 2 Polynomial
/METHOD=SSTYPE(3)
/EMMEANS=TABLES(Group*Time)
/PRINT=DESCRIPTIVE ETASQ
/CRITERIA=ALPHA(.05)
/WSDESIGN=Time
/DESIGN=Group.

**5. One-Way ANOVA (Gain Scores)**

ONEWAY Gain_Overall BY Group
/POSTHOC=TUKEY
/STATISTICS DESCRIPTIVES.

**6. SRL Reliability Analysis**

RELIABILITY
/VARIABLES=SRL_1 TO SRL_35
/SCALE('SRL_Total') ALL
/MODEL=ALPHA.

**7. Compute SRL Composite Score**

COMPUTE SRL_Total = MEAN(SRL_1 TO SRL_35).
EXECUTE.

**8. Moderation Analysis (Optional)**

UNIANOVA Gain_Overall BY Group Proficiency
/METHOD=SSTYPE(3)
/INTERCEPT=INCLUDE
/PRINT=DESCRIPTIVE ETASQ
/CRITERIA=ALPHA(.05)
/DESIGN=Group Proficiency Group*Proficiency.
