# Supplementary material for: Sustainable L2 writing pedagogy in Turkish higher education: Effects of AI-mediated feedback on self-regulated learning and writing performance
Source: PLoS One. 2026 Jul 14;21(7):e0344618. doi: 10.1371/journal.pone.0344618 (PMC13367666; doi:10.1371/journal.pone.0344618)
Supplement: S4 File — R script used for data preparation, statistical analyses, and generation of figures and supplementary outputs reported in the study. (DOCX) [file pone.0344618.s005.docx]

S4 File. R Script for Reproducible Analysis

1. Install and Load Required Packages

install.packages(c("tidyverse", "psych", "car", "emmeans", "ggplot2"))

library(tidyverse)
library(psych)
library(car)
library(emmeans)
library(ggplot2)

2. Load Dataset

data <- read.csv("PLoS_dataset_simulated.csv")

If using Excel:

library(readxl)

data <- read_excel("PLoS_dataset_simulated.xlsx")

3. Inspect Data

str(data)
summary(data)

4. Compute Gain Scores

data <- data %>%
mutate(Gain_Overall = Post_Overall - Pre_Overall)

5. Descriptive Statistics

data %>%
group_by(Group) %>%
summarise(
Mean_Pre = mean(Pre_Overall),
Mean_Post = mean(Post_Overall),
SD_Pre = sd(Pre_Overall),
SD_Post = sd(Post_Overall)

6. Mixed ANOVA (Time × Group)

library(ez)

install.packages("ez")
library(ez)

data_long <- data %>%
pivot_longer(cols = c(Pre_Overall, Post_Overall),
names_to = "Time",
values_to = "Score")

data_long$Time <- factor(data_long$Time,
levels = c("Pre_Overall", "Post_Overall"))

anova_results <- ezANOVA(
data = data_long,
dv = Score,
wid = ID,
within = .(Time),
between = .(Group),
type = 3,
detailed = TRUE)

print(anova_results)

7. One-Way ANOVA (Gain Scores)

anova_gain <- aov(Gain_Overall ~ Group, data = data)
summary(anova_gain)

8. Post-hoc Test (Tukey)

TukeyHSD(anova_gain)

9. Effect Size

library(effectsize)
eta_squared(anova_gain)

10. Reliability Analysis (SRL Scale)

srl_items <- data %>%
select(starts_with("SRL_"))

alpha(srl_items)

11. Compute SRL Composite Score

data <- data %>%
mutate(SRL_Total = rowMeans(select(., starts_with("SRL_"))))

12. Moderation Analysis (Group × Proficiency)

model_mod <- aov(Gain_Overall ~ Group * Proficiency, data = data)
summary(model_mod)

13. Visualization

ggplot(data, aes(x = Group, y = Gain_Overall, fill = Group)) +
geom_boxplot() +
theme_minimal() +
labs(title = "Gain Scores by Group",
x = "Group",
y = "Gain Score")

14. Save Results

write.csv(data, "processed_data.csv", row.names = FALSE)
