# Supplementary material for: Sustainable L2 writing pedagogy in Turkish higher education: Effects of AI-mediated feedback on self-regulated learning and writing performance
Source: PLoS One. 2026 Jul 14;21(7):e0344618. doi: 10.1371/journal.pone.0344618 (PMC13367666; doi:10.1371/journal.pone.0344618)
Supplement: S5 File — SEM analysis script and model specifications used for supplementary structural equation modeling procedures reported in the study. (DOCX) [file pone.0344618.s006.docx]

**S5 File. Structural Equation Modeling (SEM) Script – R (lavaan)**

1. Install and Load Packages

install.packages(c("lavaan", "semTools", "psych", "tidyverse"))

library(lavaan)
library(semTools)
library(psych)
library(tidyverse)

2. Load Dataset

data <- read.csv("PLoS_dataset_simulated.csv")

For Excel:

library(readxl)

data <- read_excel("PLoS_dataset_simulated.xlsx")

3. Prepare Composite Variables (if needed)

data <- data %>%
mutate(
ABE = scale(Pre_Overall), # proxy (replace with real ABE items if available)
ENG = scale(rowMeans(select(., starts_with("SRL_1"):starts_with("SRL_10")))),
SAT = scale(rowMeans(select(., starts_with("SRL_11"):starts_with("SRL_20")))),
LOY = scale(rowMeans(select(., starts_with("SRL_21"):starts_with("SRL_28")))),
REV = scale(rowMeans(select(., starts_with("SRL_29"):starts_with("SRL_35"))))
)

4. Specify SEM Model (H1–H6)

model <- '

Direct effects

ENG ~ a*ABE
SAT ~ b*ENG
LOY ~ c*ENG
REV ~ d*SAT

Indirect (mediation) effects

indirect_SAT := a*b
indirect_LOY := a*c

Total effects

total_SAT := a*b
total_LOY := a*c

5. Fit the Model

fit <- sem(model, data = data, estimator = "MLR")

6. Model Summary

summary(fit, fit.measures = TRUE, standardized = TRUE, rsquare = TRUE)

7. Fit Indices

fitMeasures(fit, c("chisq", "df", "cfi", "tli", "rmsea", "srmr"))

8. Standardized Path Coefficients

standardizedSolution(fit)

9. Mediation Effects (Bootstrapping)

fit_boot <- sem(model,
data = data,
se = "bootstrap",
bootstrap = 5000)

parameterEstimates(fit_boot,
boot.ci.type = "perc",
standardized = TRUE)

10. Visualize SEM Model (Optional)

install.packages("semPlot")
library(semPlot)

semPaths(fit,
what = "std",
layout = "tree",
edge.label.cex = 1.2,
sizeMan = 6,
sizeLat = 8)

11. Save Output

write.csv(parameterEstimates(fit),
"SEM_results.csv",
row.names = FALSE)
