# Supplementary material for: Sustainable L2 writing pedagogy in Turkish higher education: Effects of AI-mediated feedback on self-regulated learning and writing performance
Source: PLoS One. 2026 Jul 14;21(7):e0344618. doi: 10.1371/journal.pone.0344618 (PMC13367666; doi:10.1371/journal.pone.0344618)
Supplement: Attachment — Submitted filename: Response to Academic Editor and Reviewers.docx [file pone.0344618.s008.docx]

Response to Academic Editor and Reviewers

| 1. When submitting your revision, we need you to address these additional requirements.  Please ensure that your manuscript meets PLOS ONE's style requirements, including those for file naming. The PLOS ONE style templates can be found at  <https://journals.plos.org/plosone/s/file?id=wjVg/PLOSOne_formatting_sample_main_body.pdf> and  [https://journals.plos.org/plosone/s/file?id=ba62/PLOSOne_formatting_sample_title_authors_affiliations.pdf](https://track.editorialmanager.com/CL0/https:%2F%2Fjournals.plos.org%2Fplosone%2Fs%2Ffile%3Fid=ba62%2FPLOSOne_formatting_sample_title_authors_affiliations.pdf/1/010f019d608aa976-3fc5c52b-011f-4583-831b-c83c9abd59c6-000000/yhUwSkr1aqpm7Ms22yAsuJvGuGL0AUexv14S2_5J8h8=257) | Thank you, it has been revised accordingly. |
| --- | --- |
| 2. Please note that PLOS One has specific guidelines on code sharing for submissions in which author-generated code underpins the findings in the manuscript. In these cases, we expect all author-generated code to be made available without restrictions upon publication of the work. Please review our guidelines at https://journals.plos.org/plosone/s/materials-and-software-sharing#loc-sharing-code and ensure that your code is shared in a way that follows best practice and facilitates reproducibility and reuse. | Thank you for highlighting the PLOS ONE guidelines on code sharing. We confirm that all author-generated code underpinning the findings in this manuscript will be made fully and freely available upon publication.  Specifically, all statistical analysis scripts, including R scripts (for data processing, confirmatory factor analysis, structural equation modeling, and mediation analysis) and SPSS syntax files, will be shared without restriction. These materials will be provided alongside the dataset and codebook.  The code has been prepared following best practices to facilitate reproducibility and reuse. This includes clear annotation, step-by-step documentation, and alignment with the variables and procedures described in the Methods section.  No proprietary or restricted software dependencies prevent access to the shared code. All scripts can be executed using standard statistical software.  We confirm our full compliance with PLOS ONE’s materials and software sharing policy. |
| 3. We note that your Data Availability Statement is currently as follows: “All relevant data are within the manuscript and its Supporting Information files.”  Please confirm at this time whether or not your submission contains all raw data required to replicate the results of your study. Authors must share the “minimal data set” for their submission. PLOS defines the minimal data set to consist of the data required to replicate all study findings reported in the article, as well as related metadata and methods (<https://journals.plos.org/plosone/s/data-availability#loc-minimal-data-set-definition>).  For example, authors should submit the following data:  - The values behind the means, standard deviations and other measures reported;  - The values used to build graphs;  - The points extracted from images for analysis.  Authors do not need to submit their entire data set if only a portion of the data was used in the reported study.  If your submission does not contain these data, please either upload them as Supporting Information files or deposit them to a stable, public repository and provide us with the relevant URLs, DOIs, or accession numbers. For a list of recommended repositories, please see <https://journals.plos.org/plosone/s/recommended-repositories>.  If there are ethical or legal restrictions on sharing a de-identified data set, please explain them in detail (e.g., data contain potentially sensitive information, data are owned by a third-party organization, etc.) and who has imposed them (e.g., an ethics committee). Please also provide contact information for a data access committee, ethics committee, or other institutional body to which data requests may be sent. If data are owned by a third party, please indicate how others may request data access. | We will upload the complete anonymized dataset, including all individual-level data points underlying the reported analyses, as Supporting Information files upon revision. |
| 4. We notice that your supplementary figures are uploaded with the file type 'Figure'. Please amend the file type to 'Supporting Information'. Please ensure that each Supporting Information file has a legend listed in the manuscript after the references list. | Thank you for your guidance. We have inserted tables and figures in the text and also uploaded it as a supporting information |
| 5. When completing the data availability statement of the submission form, you indicated that you will make your data available on acceptance. We strongly recommend all authors decide on a data sharing plan before acceptance, as the process can be lengthy and hold up publication timelines. Please note that, though access restrictions are acceptable now, your entire data will need to be made freely accessible if your manuscript is accepted for publication. This policy applies to all data except where public deposition would breach compliance with the protocol approved by your research ethics board. If you are unable to adhere to our open data policy, please kindly revise your statement to explain your reasoning and we will seek the editor's input on an exemption. Please be assured that, once you have provided your new statement, the assessment of your exemption will not hold up the peer review process. | Thank you for your guidance regarding the data availability policy. We confirm that we have now finalized our data sharing plan in line with PLOS ONE requirements.  All data underlying the findings of this study will be made fully and freely available upon acceptance. The complete anonymized dataset, including all individual-level data required to replicate the reported results, will be provided either as Supporting Information files  The dataset does not contain any personally identifiable information, and therefore no ethical or legal restrictions apply to data sharing. A detailed codebook and analysis scripts will also be provided to ensure full reproducibility of the study.  We appreciate the importance of open science practices and confirm our full compliance with the PLOS data availability policy. |
| 6. Please include your full ethics statement in the ‘Methods’ section of your manuscript file. In your statement, please include the full name of the IRB or ethics committee who approved or waived your study, as well as whether or not you obtained informed written or verbal consent. If consent was waived for your study, please include this information in your statement as well. | The ethics statement has been revised as: “This study was conducted in accordance with the ethical standards of the Declaration of Helsinki. Ethical approval for the research was obtained from the Scientific Research and Publication Ethics Committee of Ankara Hacı Bayram Veli University. The committee reviewed and approved the study protocol, including the data collection procedures and instruments. Participation in the study was voluntary. Prior to data collection, all participants were informed about the purpose of the research, the nature of their involvement, and their rights as participants. Informed consent was obtained from all participants before participation. No personally identifiable information was collected, and all responses were recorded anonymously. Participants were informed that they could withdraw from the study at any time without any consequences. The collected data were used solely for academic research purposes and were stored securely in accordance with institutional data protection guidelines.” |
| 7. We note you have included a table to which you do not refer in the text of your manuscript. Please ensure that you refer to Table 1 in your text; if accepted, production will need this reference to link the reader to the Table. | Thank you for your attention. It has been referred in the text |

Response to Reviewer 1

| 1. Operationalization of the "Instructor-Mediated Feedback" (IMF) Condition The Issue: The description of the IMF group is the weakest point in the methodology. It is currently defined only by what it is not ("without automated feedback"). For the findings to be interpretable, readers need a clear, detailed description of what this control condition did receive.  Suggested Revision: Please provide a detailed account of the IMF condition. Crucially, what kind of feedback did these students receive from the instructor? Was it holistic scores only (as implied on Page 12, line 136), written marginal comments, a general rubric, or whole-class oral feedback? How often did they receive it? Without this, it's impossible to know if the observed effects are due to the presence of AI or the absence of meaningful, individualized instructor feedback. This is critical for the study's internal validity and for positioning the findings in the broader literature. Check this study: A systematic review of ChatGPT for English as a foreign language writing: Opportunities, challenges, and recommendations. | Thank you for this important and insightful comment. We agree that a more detailed operationalization of the IMF condition is essential for ensuring the interpretability and internal validity of the study.  In response, we have substantially revised the Method section to provide a clearer and more comprehensive description of the IMF condition. Specifically, we now clarify that students in this group received CEFR-aligned holistic scores accompanied by brief whole-class oral feedback addressing common writing issues, but did not receive individualized written comments, marginal annotations, or opportunities for draft revision. We also specify the frequency and format of feedback delivery.  Importantly, we have explicitly positioned the IMF condition as a **pedagogically realistic baseline**, reflecting prevalent feedback practices in Turkish higher education contexts characterized by large class sizes and limited time for individualized instruction. This clarification allows readers to better interpret the comparative effects of AI-mediated feedback relative to authentic instructional conditions rather than an artificially constructed control group. We have incorporated these revisions in the manuscript. Additionally, we have strengthened the theoretical framing by aligning this operationalization with prior literature on feedback scalability and constraints in EFL contexts, including recent discussions on AI-supported feedback in L2 writing. We appreciate this suggestion, which has significantly improved the clarity and rigor of our study. |
| --- | --- |
| 2. Confirmation of the "No AI-Mediated Feedback" Condition The Issue: The study took place over a 10-week intervention in a real-world university setting. It is highly plausible that students in the IMF group, despite being instructed not to, may have independently used freely available AI tools like Grammarly or ChatGPT for their writing tasks.  Suggested Revision: How did the researchers ensure or control for this threat to internal validity? Was any self-report data collected post-intervention to check for this? A brief mention of how this potential confound was addressed (e.g., "Students in all groups were asked to report any additional tools used, and a post-hoc check revealed no significant cross-contamination") would significantly strengthen the study's claims. | Thank you for raising this important concern regarding potential cross-contamination across experimental conditions. We fully agree that uncontrolled use of external AI tools could compromise the internal validity of the study.  To address this issue, we have clarified the procedural controls implemented during the intervention. Specifically, all writing tasks were completed under controlled classroom conditions without access to external digital tools. Participants were explicitly instructed not to use AI-based feedback tools outside the designated experimental conditions. In addition, we conducted a **post-intervention self-report check** in which students indicated whether they had used any external feedback tools during the study period. The results did not reveal any systematic or significant use of unauthorized AI tools within the IMF group, nor evidence of cross-group contamination. We acknowledge that, as in all naturalistic classroom research, the possibility of unreported tool use cannot be entirely eliminated. However, the combination of controlled task conditions, explicit instructions, and post-hoc verification provides reasonable assurance that the observed effects are attributable to the intended feedback interventions. This clarification has been added to the Method section to strengthen the transparency and internal validity of the study. |
| 3. Literature understanding of metacognition and regulation. Check the following articles how to improve self-regulation in AI. 1. Understanding EFL student writers’ metacognitive awareness in utilizing ChatGPT. 2. Metacognitive Awareness and EFL Learners' Writing Performance in ChatGPT‐Assisted Writing Context. 3. Metacognitive awareness and EFL learners' perceptions and experiences in utilizing ChatGPT for writing feedback | Thank you for this insightful suggestion. In response, we have strengthened the theoretical framing of metacognitive awareness and self-regulation in AI-assisted writing contexts. Specifically, we have incorporated recent empirical studies examining how learners engage with ChatGPT-mediated feedback and how such interaction fosters metacognitive processes, including planning, monitoring, and evaluation. A new paragraph has been added to the Introduction section to explicitly connect generative AI feedback with metacognitive scaffolding and self-regulated learning development. This addition clarifies the mechanism through which AI-mediated feedback supports SRL and aligns our study more closely with emerging literature on AI-supported metacognition in L2 writing. |
| 3. The Sustainability Argument The Issue: The paper's framing around "sustainable" pedagogy is a key strength. However, the argument is currently focused on learner sustainability (autonomy, lifelong learning). While important, sustainability in higher education also encompasses instructor and institutional workload.  Suggested Revision: The manuscript could be significantly strengthened by explicitly discussing the instructor workload implications. The abstract and conclusion mention "reducing instructor workload," but the data doesn't directly support this claim. The discussion (Page 29, line 450) mentions scalability "without increasing instructor workload." Consider addressing this more concretely. For example: Did the instructor spend less time on the GenAI-F group? Could the freed-up time be reallocated to higher-value pedagogical activities? Adding a note about this as a crucial area for future research would ground the sustainability argument in a more practical, institutional reality. | Thank you for this valuable observation. We agree that sustainability in higher education should encompass not only learner autonomy but also instructor workload and institutional feasibility. In response, we have revised the manuscript to clarify that instructor workload reduction was not directly measured in the present study. Specifically, we have modified the Abstract and Conclusion to avoid overgeneralization and added a new paragraph to the Discussion section that critically reflects on the potential, yet unmeasured, role of AI-mediated feedback in redistributing instructional effort. This addition elaborates how generative AI may support scalable feedback practices while enabling instructors to focus on higher-value pedagogical activities. We have also highlighted the need for future research employing time-tracking and mixed-method designs to empirically examine workload implications. These revisions strengthen the sustainability argument by grounding it in both pedagogical and institutional realities. |
| 4. Clarification of Proficiency Moderation Analysis The Issue: The moderation analysis is a valuable part of the study. However, the description of how CEFR levels were assigned is missing from the Methods section. | Thank you for highlighting this important issue. We have revised the Methods section to provide a clearer description of how CEFR proficiency levels were assigned. Specifically, we now explain how EF Standard English Test scores were converted into CEFR categories using established equivalency guidelines, including the score ranges used to classify participants into intermediate (B1–B2) and advanced (B2–C1) groups. This clarification has been added to the Participants section to enhance methodological transparency and replicability. |

Response to Reviewer 2

| The authors claimed that the capacity of automated feedback "to address higher-order such as content development, organization, and argumentation remains limited." I am not quite sure if this is correct. Since the emergence of GenAI tools, many studies have been conducted, including automated corrective feedback, and many more. Please see a recent study by Luo and Yusuf (2025), Alharbi (2023), and related ones. You may wish to acknowledge the presence of these, but critically discuss what they lack that you want to advance.  Ref:  Luo, L., & Yusuf, A. (2025). Bridging AI and pedagogy: how AI-adaptive feedback shapes Chinese EFL students’ writing engagement, metacognitive writing strategies, and writing performance. Assessment & Evaluation in Higher Education, 1–24. <https://doi.org/10.1080/02602938.2025.2548919>  Alharbi, W. 2023. “AI in the Foreign Language Classroom: A Pedagogical Overview of Automated Writing Assistance Tools.” Education Research International 2023: 1–15. <https://doi.org/10.1155/2023/4253331>. | Thank you for this insightful comment. We agree that recent advances in AI-driven feedback systems have expanded their capacity to address higher-order aspects of writing, including content development, organization, and argumentation. In response, we have revised the Introduction to acknowledge emerging evidence from recent studies (e.g., Alharbi, 2023; Luo & Yusuf, 2025) demonstrating the evolving capabilities of AI-assisted feedback. At the same time, we have refined our argument to emphasize that, despite these advances, important questions remain regarding learners’ metacognitive engagement, the sustainability of feedback use, and the development of self-regulated writing strategies. This revision strengthens the theoretical positioning of our study and clarifies its contribution within the current AI-assisted writing literature. |
| --- | --- |
| In the introduction section, the author clearly highlights how AF and GenAI-F can enhance L2 writing. They further critique AF, arguing that it relies on predefined algorithms and therefore cannot address higher-order concerns. Building on this, they explain that GenAI-F can support ideation. With this, the distinction between what both can do is already clear. My question, then, is: why do Research Questions 1 and 2 matter if we already know this difference? | Thank you for this insightful observation. We agree that prior research has already established general functional differences between automated feedback and generative AI feedback. In response, we have strengthened the rationale for our research questions in the Introduction by clarifying that the purpose of this study is not to reconfirm these differences, but to examine how they operate within a comparative classroom-based framework. Specifically, we emphasize three gaps: (1) the limited number of studies directly comparing feedback modalities under authentic instructional conditions, (2) the lack of research examining their differential impact on self-regulated learning processes, and (3) the underexplored role of learner characteristics such as proficiency. We have also slightly refined the wording of the research questions to highlight their focus on developmental and differential effects. These revisions clarify the study’s contribution beyond established distinctions in the literature. |
| In fact, the claim that AF cannot address higher-order concerns appears somewhat exaggerated. A recent meta-analysis (see Fleckenstein et al.) reports a moderate overall effect (g = 0.55) of automated feedback on students’ writing performance, suggesting that its impact may extend beyond surface-level corrections. In addition, a study by Bellhäuser and colleagues found that AF enhances better average goal setting, planning, and adherence to time schedule, as well as higher self-efficacy and satisfaction.  Ref: Bellhäuser H, Dignath C and Theobald M (2023). Daily automated feedback enhances self-regulated learning: a longitudinal randomized field experiment. Front. Psychol. 14:1125873. doi: 10.3389/fpsyg.[2023.1125873](callto:2023.1125873)  Fleckenstein J, Liebenow LW, Meyer J. Automated feedback and writing: a multi-level meta-analysis of effects on students' performance. Front Artif Intell. 2023 Jul 3;6:1162454. doi: 10.3389/frai.[2023.1162454](callto:2023.1162454). | We have revised the Introduction to moderate our earlier claim regarding the limitations of automated feedback. Specifically, we now incorporate recent evidence, including the meta-analysis by Fleckenstein et al. (2023) and the experimental study by Bellhäuser et al. (2023), which demonstrate that automated feedback can support writing performance and certain dimensions of self-regulated learning. At the same time, we clarify that questions remain regarding the depth of metacognitive engagement and the sustainability of such improvements. This revision ensures a more balanced and up-to-date representation of the literature while maintaining the rationale for our study. |
| I recommend that the authors clearly show what this study is contributing to the literature, given that many studies have found enhanced writing performance and self-regulated learning. | Thank you for this valuable suggestion. While the manuscript already outlined key research gaps, we have now strengthened the articulation of the study’s contribution by explicitly stating how it advances the literature. Specifically, we added a short contribution paragraph in the Introduction highlighting the study’s comparative design, its focus on self-regulated learning processes, and the inclusion of proficiency as a moderating variable. These revisions clarify the added value of the study beyond existing research on AI-mediated feedback. |
| There appears to be a discrepancy in the study timeline. The manuscript states that instruction was delivered over a 14-week semester, yet data were collected over 10 weeks. It is unclear how these two timeframes align. The authors should clarify whether data collection covered only a portion of the instructional period and specify the exact timing of measurements. | Thank you for this important observation. We have revised the Methods section to clarify the relationship between the 14-week instructional period and the 10-week data collection phase. Specifically, we now explain that the first two weeks were dedicated to orientation and pre-test administration, the following ten weeks constituted the main intervention and data collection period, and the final two weeks were used for post-test administration and course completion. This clarification has been added to the Procedure section to ensure transparency and replicability. |
| The description of the writing task is somewhat limited. It is unclear whether the task involved integrated writing (e.g., based on source texts) or independent writing. In addition, the essay prompt or topic should be provided for transparency, as this is essential for understanding task demands and interpreting the findings. | Thank you for this valuable suggestion. We have revised the Methods section to clarify the nature of the writing tasks. Specifically, we now indicate that the tasks were independent argumentative writing tasks rather than integrated, source-based tasks. In addition, we have provided an example of the essay prompt and described the general characteristics of the topics to enhance transparency and replicability. These revisions help clarify task demands and support a more accurate interpretation of the findings. |
| The methodology section is quite dense, which affects readability. I recommend structuring into subsections such as design and participants, research context, instruments, procedure, and data analysis. | Thank you for this helpful suggestion. In response, we have reorganized the Methodology section into clearly defined subsections (Research Context, Research Design and Participants, Data Collection, Data Collection Tools and Data Analysis) without altering the original content. This restructuring improves readability and aligns the manuscript with standard reporting practices. |
| The use of ChatGPT-4 for initial essay scoring raises some concerns. While the L2 Composition Profile is an established rubric, ChatGPT itself is not a validated scoring instrument, and the majority of essays appear to have been rated solely by the AI system. Although interrater reliability with a human rater is reported for a subset of scripts, this may not be sufficient to ensure scoring validity across the full dataset. In addition, given that generative AI is also used as an intervention tool in the study, the potential for methodological bias should be more explicitly acknowledged and addressed. | Thank you for raising this important concern. We agree that the use of ChatGPT-4 as part of the scoring process requires careful clarification. In response, we have revised the Methods section to explicitly state that ChatGPT was used as a rubric-guided evaluation assistant rather than as an scoring instrument. We have also clarified that all scoring was based on the L2 Composition Profile and that interrater reliability was established through independent human rating of a subset of scripts (ICC = 0.81).  Furthermore, we have acknowledged the limitation that the majority of essays were scored through this AI-assisted procedure and explicitly discussed the potential for methodological bias, given the dual role of generative AI in both instruction and assessment. This limitation has now been addressed in the Discussion section, along with suggestions for future research incorporating fully independent human rating procedures. These revisions improve the transparency and methodological rigor of the study. |
| The study does not provide sufficient evidence regarding treatment fidelity or actual engagement with the feedback tools. It remains unclear how consistently and effectively participants used Grammarly and ChatGPT, which may influence the observed effects. | Thank you for this important point. We have revised the Methodology section to clarify how treatment fidelity and engagement were supported and monitored. Specifically, we now describe the structured training provided to the AF and GenAI-F groups, in-class monitoring of tool use, and the use of brief post-task checklists to confirm engagement with feedback. We also acknowledge that the study did not collect fine-grained usage data (e.g., interaction logs or time-on-task) and that variability in individual engagement cannot be fully ruled out. This limitation has been explicitly noted in the Discussion, along with recommendations for future research using process-tracing methods. |
| The choice of analytical approach raises concerns about its alignment with the research questions. The use of a mixed-design ANOVA suggests an interest in changes over time and interaction effects. However, RQ1 and RQ2 are framed in terms of differences between feedback types, without explicitly referencing change over time. If the intention is to examine improvement, this should be made clearer in the research questions. Alternatively, if the focus is on post-test differences, MANCOVA or ANCOVA will be more appropriate to control for baseline performance. | Thank you for this insightful observation. We agree that the research questions should more clearly reflect the longitudinal nature of the analysis. In response, we have revised RQ1 and RQ2 to explicitly focus on changes over time, consistent with the mixed-design ANOVA employed in the study. We have also added a clarifying statement in the manuscript to emphasize that the analysis examines both within-subject development and between-group differences. These revisions ensure better alignment between the research questions, study design, and analytical approach. |
| In fact, the authors claimed that Bonferroni was computed to control inflated Type I error, but then went ahead to run separate ANOVAs instead of MANOVA or MANCOVA, as the case may be. The manuscript reports Bonferroni-adjusted pairwise comparisons even in cases where the corresponding main or interaction effects are non-significant (see Table 3). This raises concerns about the appropriateness of these post hoc tests, as they are generally warranted only following significant omnibus effects. | Thank you for this important methodological observation. We acknowledge that the use of multiple univariate ANOVAs, rather than a multivariate approach, may increase the risk of Type I error. In response, we have clarified in the Data Analysis section that separate ANOVAs were employed to allow dimension-specific interpretation of self-regulated learning strategies, which are theoretically distinct constructs. We have also explicitly noted this as a limitation and suggested the use of MANOVA/MANCOVA in future research.  Additionally, we have revised the Results section to clarify that post hoc comparisons are interpreted only when corresponding omnibus effects are statistically significant. Where post hoc results are presented for completeness despite non-significant omnibus tests, they are now explicitly described as exploratory and interpreted with caution. These revisions improve the transparency and statistical rigor of the manuscript. |
| The overall result section is too dense, and I often miss which analysis is addressing which research question. I recommend re-stating the research question or appropriate headings before presenting the corresponding results. | Thank you for this helpful suggestion. We have revised the Results section to improve clarity by explicitly aligning each set of analyses with the corresponding research questions. Specifically, we added subsection headings (RQ1, RQ2, RQ3) and brief introductory sentences to guide the reader through the structure of the findings. These revisions enhance readability and make it easier to follow how each analysis addresses the study’s research questions. |
| There appears to be an inconsistency in the analytical approach for RQ3. While earlier analyses incorporate time to capture development, the use of a two-way ANOVA here excludes the temporal dimension, despite the research question referring to writing development. Clarification is needed regarding whether development is operationalised as change over time or as post-test performance. | Thank you for this insightful observation. We agree that clarification is needed regarding how development is operationalized across the research questions. In response, we have revised the manuscript to explicitly distinguish between the longitudinal analyses (RQ1 and RQ2), which examine changes over time, and the moderation analysis (RQ3), which focuses on post-test outcomes. We have also updated the wording of RQ3 to reflect this distinction and added clarifying statements in both the Data Analysis and Results sections. These revisions ensure consistency between the research questions, analytical approach, and interpretation of findings. |
| Some interpretations appear to extend beyond the statistical results, particularly where non-significant interaction effects are discussed as indicative of differential impact. Greater caution in interpreting non-significant findings is recommended. | Thank you for this important observation. We agree that interpretations of non-significant findings should be treated with greater caution. In response, we have revised the Results and Discussion sections to ensure that claims regarding differential effects are based only on statistically significant results. Where descriptive differences are reported, they are now explicitly framed as non-significant trends and interpreted cautiously. We have also added a clarifying statement at the beginning of the Results section to guide interpretation. These revisions improve the accuracy and statistical rigor of the manuscript. |
|  |  |
