# Supplementary material for: Sustainable L2 writing pedagogy in Turkish higher education: Effects of AI-mediated feedback on self-regulated learning and writing performance
Source: PLoS One. 2026 Jul 14;21(7):e0344618. doi: 10.1371/journal.pone.0344618 (PMC13367666; doi:10.1371/journal.pone.0344618)
Supplement: Peer review history

# Peer review: Decision Letter 0

**DOI:** 10.1371/journal.pone.0344618.r001

**Authors:** Wei Lun Wong

5 Apr 2026

PONE-D-26-07893

Sustainable L2 Writing Pedagogy in Turkish Higher Education: Effects of AI-Mediated Feedback on Self-Regulated Learning and Writing Performance

PLOS One

Dear Dr.  IRGATOĞLU,

Thank you for submitting your manuscript to PLOS ONE. After careful consideration, we feel that it has merit but does not fully meet PLOS ONE’s publication criteria as it currently stands. Therefore, we invite you to submit a revised version of the manuscript that addresses the points raised during the review process.

Please submit your revised manuscript by May 20 2026 11:59PM. If you will need more time than this to complete your revisions, please reply to this message or contact the journal office at plosone@plos.org. When you're ready to submit your revision, log on to https://www.editorialmanager.com/pone/ and select the 'Submissions Needing Revision' folder to locate your manuscript file.

Please include the following items when submitting your revised manuscript:

- A letter that responds to each point raised by the academic editor and reviewer(s). You should upload this letter as a separate file labeled 'Response to Reviewers'.
- A marked-up copy of your manuscript that highlights changes made to the original version. You should upload this as a separate file labeled 'Revised Manuscript with Track Changes'.
- An unmarked version of your revised paper without tracked changes. You should upload this as a separate file labeled 'Manuscript'.

If you would like to make changes to your financial disclosure, please include your updated statement in your cover letter. Guidelines for resubmitting your figure files are available below the reviewer comments at the end of this letter.

If applicable, we recommend that you deposit your laboratory protocols in protocols.io to enhance the reproducibility of your results. Protocols.io assigns your protocol its own identifier (DOI) so that it can be cited independently in the future. For instructions see: https://journals.plos.org/plosone/s/submission-guidelines#loc-laboratory-protocols. Additionally, PLOS ONE offers an option for publishing peer-reviewed Lab Protocol articles, which describe protocols hosted on protocols.io. Read more information on sharing protocols at https://plos.org/protocols?utm_medium=editorial-email&utm_source=authorletters&utm_campaign=protocols.

As the corresponding author, your ORCID iD is verified in the submission system and will appear in the published article. PLOS supports the use of ORCID, and we encourage all coauthors to register for an ORCID iD and use it as well. Please encourage your coauthors to verify their ORCID iD within the submission system before final acceptance, as unverified ORCID iDs will not appear in the published article. *Only*  the individual author can complete the verification step; PLOS staff *cannot*  verify ORCID iDs on behalf of authors.

We look forward to receiving your revised manuscript.

Kind regards,

Wei Lun Wong

Academic Editor

PLOS One

Journal Requirements:

When submitting your revision, we need you to address these additional requirements.

Please ensure that your manuscript meets PLOS ONE's style requirements, including those for file naming. The PLOS ONE style templates can be found at

https://journals.plos.org/plosone/s/file?id=wjVg/PLOSOne_formatting_sample_main_body.pdf and

https://journals.plos.org/plosone/s/file?id=ba62/PLOSOne_formatting_sample_title_authors_affiliations.pdf

2. Please note that PLOS One has specific guidelines on code sharing for submissions in which author-generated code underpins the findings in the manuscript. In these cases, we expect all author-generated code to be made available without restrictions upon publication of the work. Please review our guidelines at https://journals.plos.org/plosone/s/materials-and-software-sharing#loc-sharing-code and ensure that your code is shared in a way that follows best practice and facilitates reproducibility and reuse.

3. We note that your Data Availability Statement is currently as follows: “All relevant data are within the manuscript and its Supporting Information files.”

Please confirm at this time whether or not your submission contains all raw data required to replicate the results of your study. Authors must share the “minimal data set” for their submission. PLOS defines the minimal data set to consist of the data required to replicate all study findings reported in the article, as well as related metadata and methods (https://journals.plos.org/plosone/s/data-availability#loc-minimal-data-set-definition).

For example, authors should submit the following data:

- The values behind the means, standard deviations and other measures reported;

- The values used to build graphs;

- The points extracted from images for analysis.

Authors do not need to submit their entire data set if only a portion of the data was used in the reported study.

If your submission does not contain these data, please either upload them as Supporting Information files or deposit them to a stable, public repository and provide us with the relevant URLs, DOIs, or accession numbers. For a list of recommended repositories, please see https://journals.plos.org/plosone/s/recommended-repositories.

If there are ethical or legal restrictions on sharing a de-identified data set, please explain them in detail (e.g., data contain potentially sensitive information, data are owned by a third-party organization, etc.) and who has imposed them (e.g., an ethics committee). Please also provide contact information for a data access committee, ethics committee, or other institutional body to which data requests may be sent. If data are owned by a third party, please indicate how others may request data access.

4. We notice that your supplementary figures are uploaded with the file type 'Figure'. Please amend the file type to 'Supporting Information'. Please ensure that each Supporting Information file has a legend listed in the manuscript after the references list.

5. When completing the data availability statement of the submission form, you indicated that you will make your data available on acceptance. We strongly recommend all authors decide on a data sharing plan before acceptance, as the process can be lengthy and hold up publication timelines. Please note that, though access restrictions are acceptable now, your entire data will need to be made freely accessible if your manuscript is accepted for publication. This policy applies to all data except where public deposition would breach compliance with the protocol approved by your research ethics board. If you are unable to adhere to our open data policy, please kindly revise your statement to explain your reasoning and we will seek the editor's input on an exemption. Please be assured that, once you have provided your new statement, the assessment of your exemption will not hold up the peer review process.

6. Please include your full ethics statement in the ‘Methods’ section of your manuscript file. In your statement, please include the full name of the IRB or ethics committee who approved or waived your study, as well as whether or not you obtained informed written or verbal consent. If consent was waived for your study, please include this information in your statement as well.

7. We note you have included a table to which you do not refer in the text of your manuscript. Please ensure that you refer to Table 1 in your text; if accepted, production will need this reference to link the reader to the Table.

8. If the reviewer comments include a recommendation to cite specific previously published works, please review and evaluate these publications to determine whether they are relevant and should be cited. There is no requirement to cite these works unless the editor has indicated otherwise.

[Note: HTML markup is below. Please do not edit.]

Reviewer's Responses to Questions

**Comments to the Author**

1. Is the manuscript technically sound, and do the data support the conclusions?

The manuscript must describe a technically sound piece of scientific research with data that supports the conclusions. Experiments must have been conducted rigorously, with appropriate controls, replication, and sample sizes. The conclusions must be drawn appropriately based on the data presented.

Reviewer #1: Yes

Reviewer #2: Yes

**********

2. Has the statistical analysis been performed appropriately and rigorously?

Reviewer #1: Yes

Reviewer #2: No

**********

3. Have the authors made all data underlying the findings in their manuscript fully available?

The PLOS Data policy requires authors to make all data underlying the findings described in their manuscript fully available without restriction, with rare exception (please refer to the Data Availability Statement in the manuscript PDF file). The data should be provided as part of the manuscript or its supporting information, or deposited to a public repository. For example, in addition to summary statistics, the data points behind means, medians and variance measures should be available. If there are restrictions on publicly sharing data—e.g. participant privacy or use of data from a third party—those must be specified.

Reviewer #1: Yes

Reviewer #2: Yes

**********

4. Is the manuscript presented in an intelligible fashion and written in standard English?

PLOS ONE does not copyedit accepted manuscripts, so the language in submitted articles must be clear, correct, and unambiguous. Any typographical or grammatical errors should be corrected at revision, so please note any specific errors here.

Reviewer #1: Yes

Reviewer #2: Yes

**********

5. Review Comments to the Author

Please use the space provided to explain your answers to the questions above. You may also include additional comments for the author, including concerns about dual publication, research ethics, or publication ethics. (Please upload your review as an attachment if it exceeds 20,000 characters)

Reviewer #1: This manuscript reports on a quasi-experimental study investigating the effects of automated feedback (Grammarly), generative AI feedback (ChatGPT), and instructor-mediated feedback on Turkish EFL learners' SRL strategies and writing performance. The study is methodologically sound, the analysis is thorough, and the findings are theoretically and pedagogically significant. However, several areas require clarification and revision to enhance the manuscript's clarity, transparency, and overall impact.

Major Comments

1. Operationalization of the "Instructor-Mediated Feedback" (IMF) Condition

The Issue: The description of the IMF group is the weakest point in the methodology. It is currently defined only by what it is not ("without automated feedback"). For the findings to be interpretable, readers need a clear, detailed description of what this control condition did receive.

Suggested Revision: Please provide a detailed account of the IMF condition. Crucially, what kind of feedback did these students receive from the instructor? Was it holistic scores only (as implied on Page 12, line 136), written marginal comments, a general rubric, or whole-class oral feedback? How often did they receive it? Without this, it's impossible to know if the observed effects are due to the presence of AI or the absence of meaningful, individualized instructor feedback. This is critical for the study's internal validity and for positioning the findings in the broader literature. Check this study: A systematic review of ChatGPT for English as a foreign language writing: Opportunities, challenges, and recommendations.

2. Confirmation of the "No AI-Mediated Feedback" Condition

The Issue: The study took place over a 10-week intervention in a real-world university setting. It is highly plausible that students in the IMF group, despite being instructed not to, may have independently used freely available AI tools like Grammarly or ChatGPT for their writing tasks.

Suggested Revision: How did the researchers ensure or control for this threat to internal validity? Was any self-report data collected post-intervention to check for this? A brief mention of how this potential confound was addressed (e.g., "Students in all groups were asked to report any additional tools used, and a post-hoc check revealed no significant cross-contamination") would significantly strengthen the study's claims.

3. Literature understanding of metacognition and regulation. Check the following articles how to improve self-regualtion in AI. 1. Understanding EFL student writers’ metacognitive awareness in utilizing ChatGPT. 2. Metacognitive Awareness and EFL Learners' Writing Performance in ChatGPT‐Assisted Writing Context. 3. Metacognitive awareness and EFL learners' perceptions and experiences in utilising ChatGPT for writing feedback

3. The Sustainability Argument

The Issue: The paper's framing around "sustainable" pedagogy is a key strength. However, the argument is currently focused on learner sustainability (autonomy, lifelong learning). While important, sustainability in higher education also encompasses instructor and institutional workload.

Suggested Revision: The manuscript could be significantly strengthened by explicitly discussing the instructor workload implications. The abstract and conclusion mention "reducing instructor workload," but the data doesn't directly support this claim. The discussion (Page 29, line 450) mentions scalability "without increasing instructor workload." Consider addressing this more concretely. For example: Did the instructor spend less time on the GenAI-F group? Could the freed-up time be reallocated to higher-value pedagogical activities? Adding a note about this as a crucial area for future research would ground the sustainability argument in a more practical, institutional reality.

4. Clarification of Proficiency Moderation Analysis

The Issue: The moderation analysis is a valuable part of the study. However, the description of how CEFR levels were assigned is missing from the Methods section.

Reviewer #2: I commend the authors’ effort in conducting this interesting study. The research examines the effects of automated, generative AI, and instructor-mediated feedback on L2 learners’ self-regulated learning strategies and writing performance, and reports differential gains across feedback types. However, I have some concerns regarding the conceptual framing, methodological alignment, and analytical approach.

Before proceeding to read my comments, the author should know that comments do not hold authoritative status. Therefore, they submit constructive rebuttals in instances where they disagreed, of course, with strong justifications.

The authors claimed that the capacity of automated feedback "to address higher-order such as content development, organization, and argumentation remains limited." I am not quite sure if this is correct. Since the emergence of GenAI tools, many studies have been conducted, including automated corrective feedback, and many more. Please see a recent study by Luo and Yusuf (2025), Alharbi (2023), and related ones. You may wish to acknowledge the presence of these, but critically discuss what they lack that you want to advance.

Ref:

Luo, L., & Yusuf, A. (2025). Bridging AI and pedagogy: how AI-adaptive feedback shapes Chinese EFL students’ writing engagement, metacognitive writing strategies, and writing performance. Assessment & Evaluation in Higher Education, 1–24. https://doi.org/10.1080/02602938.2025.2548919

Alharbi, W. 2023. “AI in the Foreign Language Classroom: A Pedagogical Overview of Automated Writing Assistance Tools.” Education Research International 2023: 1–15. https://doi.org/10.1155/2023/4253331.

In the introduction section, the author clearly highlights how AF and GenAI-F can enhance L2 writing. They further critique AF, arguing that it relies on predefined algorithms and therefore cannot address higher-order concerns. Building on this, they explain that GenAI-F can support ideation. With this, the distinction between what both can do is already clear. My question, then, is: why do Research Questions 1 and 2 matter if we already know this difference?

In fact, the claim that AF cannot address higher-order concerns appears somewhat exaggerated. A recent meta-analysis (see Fleckenstein et al.) reports a moderate overall effect (g = 0.55) of automated feedback on students’ writing performance, suggesting that its impact may extend beyond surface-level corrections. In addition, a study by Bellhäuser and colleagues found that AF enhances better average goal setting, planning, and adherence to time schedule, as well as higher self-efficacy and satisfaction.

Ref:

Bellhäuser H, Dignath C and Theobald M (2023). Daily automated feedback enhances self-regulated learning: a longitudinal randomized field experiment. Front. Psychol. 14:1125873. doi: 10.3389/fpsyg.2023.1125873

Fleckenstein J, Liebenow LW, Meyer J. Automated feedback and writing: a multi-level meta-analysis of effects on students' performance. Front Artif Intell. 2023 Jul 3;6:1162454. doi: 10.3389/frai.2023.1162454.

I recommend that the authors clearly show what this study is contributing to the literature, given that many studies have found enhanced writing performance and self-regulated learning.

There appears to be a discrepancy in the study timeline. The manuscript states that instruction was delivered over a 14-week semester, yet data were collected over 10 weeks. It is unclear how these two timeframes align. The authors should clarify whether data collection covered only a portion of the instructional period and specify the exact timing of measurements.

The description of the writing task is somewhat limited. It is unclear whether the task involved integrated writing (e.g., based on source texts) or independent writing. In addition, the essay prompt or topic should be provided for transparency, as this is essential for understanding task demands and interpreting the findings.

The methodology section is quite dense, which affects readability. I recommend structuring into subsections such as design and participants, research context, instruments, procedure, and data analysis.

The use of ChatGPT-4 for initial essay scoring raises some concerns. While the L2 Composition Profile is an established rubric, ChatGPT itself is not a validated scoring instrument, and the majority of essays appear to have been rated solely by the AI system. Although interrater reliability with a human rater is reported for a subset of scripts, this may not be sufficient to ensure scoring validity across the full dataset. In addition, given that generative AI is also used as an intervention tool in the study, the potential for methodological bias should be more explicitly acknowledged and addressed.

The study does not provide sufficient evidence regarding treatment fidelity or actual engagement with the feedback tools. It remains unclear how consistently and effectively participants used Grammarly and ChatGPT, which may influence the observed effects.

The choice of analytical approach raises concerns about its alignment with the research questions. The use of a mixed-design ANOVA suggests an interest in changes over time and interaction effects. However, RQ1 and RQ2 are framed in terms of differences between feedback types, without explicitly referencing change over time. If the intention is to examine improvement, this should be made clearer in the research questions. Alternatively, if the focus is on post-test differences, MANCOVA or ANCOVA will be more appropriate to control for baseline performance.

In fact, the authors claimed that Bonferroni was computed to control inflated Type I error, but then went ahead to run separate ANOVAs instead of MANOVA or MANCOVA, as the case may be.

The manuscript reports Bonferroni-adjusted pairwise comparisons even in cases where the corresponding main or interaction effects are non-significant (see Table 3). This raises concerns about the appropriateness of these post hoc tests, as they are generally warranted only following significant omnibus effects.

The overall result section is too dense, and I often miss which analysis is addressing which research question. I recommend re-stating the research question or appropriate headings before presenting the corresponding results.

There appears to be an inconsistency in the analytical approach for RQ3. While earlier analyses incorporate time to capture development, the use of a two-way ANOVA here excludes the temporal dimension, despite the research question referring to writing development. Clarification is needed regarding whether development is operationalised as change over time or as post-test performance.

Some interpretations appear to extend beyond the statistical results, particularly where non-significant interaction effects are discussed as indicative of differential impact. Greater caution in interpreting non-significant findings is recommended.

Given these concerns, I will recommend a major revision.

**********

6. PLOS authors have the option to publish the peer review history of their article (what does this mean?). If published, this will include your full peer review and any attached files.

If you choose “no”, your identity will remain anonymous but your review may still be made public.

**Do you want your identity to be public for this peer review?**  For information about this choice, including consent withdrawal, please see our Privacy Policy.

Reviewer #1: No

Reviewer #2: No

**********

[NOTE: If reviewer comments were submitted as an attachment file, they will be attached to this email and accessible via the submission site. Please log into your account, locate the manuscript record, and check for the action link "View Attachments". If this link does not appear, there are no attachment files.]

To ensure your figures meet our technical requirements, please review our figure guidelines: https://journals.plos.org/plosone/s/figures

You may also use PLOS’s free figure tool, NAAS, to help you prepare publication quality figures: https://journals.plos.org/plosone/s/figures#loc-tools-for-figure-preparation.

NAAS will assess whether your figures meet our technical requirements by comparing each figure against our figure specifications.

# Author comment: Author response to Decision Letter 1

**DOI:** 10.1371/journal.pone.0344618.r002

5 May 2026

Response to Academic Editor and Reviewers

1. When submitting your revision, we need you to address these additional requirements.

Please ensure that your manuscript meets PLOS ONE's style requirements, including those for file naming. The PLOS ONE style templates can be found at

https://journals.plos.org/plosone/s/file?id=wjVg/PLOSOne_formatting_sample_main_body.pdf and

https://journals.plos.org/plosone/s/file?id=ba62/PLOSOne_formatting_sample_title_authors_affiliations.pdf

Thank you, it has been revised accordingly.

2. Please note that PLOS One has specific guidelines on code sharing for submissions in which author-generated code underpins the findings in the manuscript. In these cases, we expect all author-generated code to be made available without restrictions upon publication of the work. Please review our guidelines at https://journals.plos.org/plosone/s/materials-and-software-sharing#loc-sharing-code and ensure that your code is shared in a way that follows best practice and facilitates reproducibility and reuse. Thank you for highlighting the PLOS ONE guidelines on code sharing. We confirm that all author-generated code underpinning the findings in this manuscript will be made fully and freely available upon publication.

Specifically, all statistical analysis scripts, including R scripts (for data processing, confirmatory factor analysis, structural equation modeling, and mediation analysis) and SPSS syntax files, will be shared without restriction. These materials will be provided alongside the dataset and codebook.

The code has been prepared following best practices to facilitate reproducibility and reuse. This includes clear annotation, step-by-step documentation, and alignment with the variables and procedures described in the Methods section.

No proprietary or restricted software dependencies prevent access to the shared code. All scripts can be executed using standard statistical software.

We confirm our full compliance with PLOS ONE’s materials and software sharing policy.

3. We note that your Data Availability Statement is currently as follows: “All relevant data are within the manuscript and its Supporting Information files.”

Please confirm at this time whether or not your submission contains all raw data required to replicate the results of your study. Authors must share the “minimal data set” for their submission. PLOS defines the minimal data set to consist of the data required to replicate all study findings reported in the article, as well as related metadata and methods (https://journals.plos.org/plosone/s/data-availability#loc-minimal-data-set-definition).

For example, authors should submit the following data:

- The values behind the means, standard deviations and other measures reported;

- The values used to build graphs;

- The points extracted from images for analysis.

Authors do not need to submit their entire data set if only a portion of the data was used in the reported study.

If your submission does not contain these data, please either upload them as Supporting Information files or deposit them to a stable, public repository and provide us with the relevant URLs, DOIs, or accession numbers. For a list of recommended repositories, please see https://journals.plos.org/plosone/s/recommended-repositories.

If there are ethical or legal restrictions on sharing a de-identified data set, please explain them in detail (e.g., data contain potentially sensitive information, data are owned by a third-party organization, etc.) and who has imposed them (e.g., an ethics committee). Please also provide contact information for a data access committee, ethics committee, or other institutional body to which data requests may be sent. If data are owned by a third party, please indicate how others may request data access.

We will upload the complete anonymized dataset, including all individual-level data points underlying the reported analyses, as Supporting Information files upon revision.

4. We notice that your supplementary figures are uploaded with the file type 'Figure'. Please amend the file type to 'Supporting Information'. Please ensure that each Supporting Information file has a legend listed in the manuscript after the references list.

Thank you for your guidance. We have inserted tables and figures in the text and also uploaded it as a supporting information

5. When completing the data availability statement of the submission form, you indicated that you will make your data available on acceptance. We strongly recommend all authors decide on a data sharing plan before acceptance, as the process can be lengthy and hold up publication timelines. Please note that, though access restrictions are acceptable now, your entire data will need to be made freely accessible if your manuscript is accepted for publication. This policy applies to all data except where public deposition would breach compliance with the protocol approved by your research ethics board. If you are unable to adhere to our open data policy, please kindly revise your statement to explain your reasoning and we will seek the editor's input on an exemption. Please be assured that, once you have provided your new statement, the assessment of your exemption will not hold up the peer review process.

Thank you for your guidance regarding the data availability policy. We confirm that we have now finalized our data sharing plan in line with PLOS ONE requirements.

All data underlying the findings of this study will be made fully and freely available upon acceptance. The complete anonymized dataset, including all individual-level data required to replicate the reported results, will be provided either as Supporting Information files

The dataset does not contain any personally identifiable information, and therefore no ethical or legal restrictions apply to data sharing. A detailed codebook and analysis scripts will also be provided to ensure full reproducibility of the study.

We appreciate the importance of open science practices and confirm our full compliance with the PLOS data availability policy.

6. Please include your full ethics statement in the ‘Methods’ section of your manuscript file. In your statement, please include the full name of the IRB or ethics committee who approved or waived your study, as well as whether or not you obtained informed written or verbal consent. If consent was waived for your study, please include this information in your statement as well. The ethics statement has been revised as: “This study was conducted in accordance with the ethical standards of the Declaration of Helsinki. Ethical approval for the research was obtained from the Scientific Research and Publication Ethics Committee of Ankara Hacı Bayram Veli University. The committee reviewed and approved the study protocol, including the data collection procedures and instruments. Participation in the study was voluntary. Prior to data collection, all participants were informed about the purpose of the research, the nature of their involvement, and their rights as participants. Informed consent was obtained from all participants before participation. No personally identifiable information was collected, and all responses were recorded anonymously. Participants were informed that they could withdraw from the study at any time without any consequences. The collected data were used solely for academic research purposes and were stored securely in accordance with institutional data protection guidelines.”

7. We note you have included a table to which you do not refer in the text of your manuscript. Please ensure that you refer to Table 1 in your text; if accepted, production will need this reference to link the reader to the Table. Thank you for your attention. It has been referred in the text

Response to Reviewer 1

1. Operationalization of the "Instructor-Mediated Feedback" (IMF) Condition

The Issue: The description of the IMF group is the weakest point in the methodology. It is currently defined only by what it is not ("without automated feedback"). For the findings to be interpretable, readers need a clear, detailed description of what this control condition did receive.

Suggested Revision: Please provide a detailed account of the IMF condition. Crucially, what kind of feedback did these students receive from the instructor? Was it holistic scores only (as implied on Page 12, line 136), written marginal comments, a general rubric, or whole-class oral feedback? How often did they receive it? Without this, it's impossible to know if the observed effects are due to the presence of AI or the absence of meaningful, individualized instructor feedback. This is critical for the study's internal validity and for positioning the findings in the broader literature. Check this study: A systematic review of ChatGPT for English as a foreign language writing: Opportunities, challenges, and recommendations.

Thank you for this important and insightful comment. We agree that a more detailed operationalization of the IMF condition is essential for ensuring the interpretability and internal validity of the study.

In response, we have substantially revised the Method section to provide a clearer and more comprehensive description of the IMF condition. Specifically, we now clarify that students in this group received CEFR-aligned holistic scores accompanied by brief whole-class oral feedback addressing common writing issues, but did not receive individualized written comments, marginal annotations, or opportunities for draft revision. We also specify the frequency and format of feedback delivery.

Importantly, we have explicitly positioned the IMF condition as a pedagogically realistic baseline, reflecting prevalent feedback practices in Turkish higher education contexts characterized by large class sizes and limited time for individualized instruction. This clarification allows readers to better interpret the comparative effects of AI-mediated feedback relative to authentic instructional conditions rather than an artificially constructed control group. We have incorporated these revisions in the manuscript. Additionally, we have strengthened the theoretical framing by aligning this operationalization with prior literature on feedback scalability and constraints in EFL contexts, including recent discussions on AI-supported feedback in L2 writing. We appreciate this suggestion, which has significantly improved the clarity and rigor of our study.

2. Confirmation of the "No AI-Mediated Feedback" Condition

The Issue: The study took place over a 10-week intervention in a real-world university setting. It is highly plausible that students in the IMF group, despite being instructed not to, may have independently used freely available AI tools like Grammarly or ChatGPT for their writing tasks.

Suggested Revision: How did the researchers ensure or control for this threat to internal validity? Was any self-report data collected post-intervention to check for this? A brief mention of how this potential confound was addressed (e.g., "Students in all groups were asked to report any additional tools used, and a post-hoc check revealed no significant cross-contamination") would significantly strengthen the study's claims.

Thank you for raising this important concern regarding potential cross-contamination across experimental conditions. We fully agree that uncontrolled use of external AI tools could compromise the internal validity of the study.

To address this issue, we have clarified the procedural controls implemented during the intervention. Specifically, all writing tasks were completed under controlled classroom conditions without access to external digital tools. Participants were explicitly instructed not to use AI-based feedback tools outside the designated experimental conditions. In addition, we conducted a post-intervention self-report check in which students indicated whether they had used any external feedback tools during the study period. The results did not reveal any systematic or significant use of unauthorized AI tools within the IMF group, nor evidence of cross-group contamination. We acknowledge that, as in all naturalistic classroom research, the possibility of unreported tool use cannot be entirely eliminated. However, the combination of controlled task conditions, explicit instructions, and post-hoc verification provides reasonable assurance that the observed effects are attributable to the intended feedback interventions. This clarification has been added to the Method section to strengthen the transparency and internal validity of the study.

3. Literature understanding of metacognition and regulation. Check the following articles how to improve self-regulation in AI. 1. Understanding EFL student writers’ metacognitive awareness in utilizing ChatGPT. 2. Metacognitive Awareness and EFL Learners' Writing Performance in ChatGPT‐Assisted Writing Context. 3. Metacognitive awareness and EFL learners' perceptions and experiences in utilizing ChatGPT for writing feedback

Thank you for this insightful suggestion. In response, we have strengthened the theoretical framing of metacognitive awareness and self-regulation in AI-assisted writing contexts. Specifically, we have incorporated recent empirical studies examining how learners engage with ChatGPT-mediated feedback and how such interaction fosters metacognitive processes, including planning, monitoring, and evaluation. A new paragraph has been added to the Introduction section to explicitly connect generative AI feedback with metacognitive scaffolding and self-regulated learning development. This addition clarifies the mechanism through which AI-mediated feedback supports SRL and aligns our study more closely with emerging literature on AI-supported metacognition in L2 writing.

3. The Sustainability Argument

The Issue: The paper's framing around "sustainable" pedagogy is a key strength. However, the argument is currently focused on learner sustainability (autonomy, lifelong learning). While important, sustainability in higher education also encompasses instructor and institutional workload.

Suggested Revision: The manuscript could be significantly strengthened by explicitly discussing the instructor workload implications. The abstract and conclusion mention "reducing instructor workload," but the data doesn't directly support this claim. The discussion (Page 29, line 450) mentions scalability "without increasing instructor workload." Consider addressing this more concretely. For example: Did the instructor spend less time on the GenAI-F group? Could the freed-up time be reallocated to higher-value pedagogical activities? Adding a note about this as a crucial area for future research would ground the sustainability argument in a more practical, institutional reality.

Thank you for this valuable observation. We agree that sustainability in higher education should encompass not only learner autonomy but also instructor workload and institutional feasibility. In response, we have revised the manuscript to clarify that instructor workload reduction was not directly measured in the present study. Specifically, we have modified the Abstract and Conclusion to avoid overgeneralization and added a new paragraph to the Discussion section that critically reflects on the potential, yet unmeasured, role of AI-mediated feedback in redistributing instructional effort. This addition elaborates how generative AI may support scalable feedback practices while enabling instructors to focus on higher-value pedagogical activities. We have also highlighted the need for future research employing time-tracking and mixed-method designs to empirically examine workload implications. These revisions strengthen the sustainability argument by grounding it in both pedagogical and institutional realities.

4. Clarification of Proficiency Moderation Analysis

The Issue: The moderation analysis is a valuable part of the study. However, the description of how CEFR levels were assigned is missing from the Methods section.

Thank you for highlighting this important issue. We have revised the Methods section to provide a clearer description of how CEFR proficiency levels were assigned. Specifically, we now explain how EF Standard English Te

Attachment Submitted filename: Response to Academic Editor and Reviewers.docx

# Peer review: Decision Letter 1

**DOI:** 10.1371/journal.pone.0344618.r003

**Authors:** Wei Lun Wong, Laura Kelly

14 Jun 2026

Sustainable L2 Writing Pedagogy in Turkish Higher Education: Effects of AI-Mediated Feedback on Self-Regulated Learning and Writing Performance

PONE-D-26-07893R1

Dear Dr. IRGATOĞLU,

We’re pleased to inform you that your manuscript has been judged scientifically suitable for publication and will be formally accepted for publication once it meets all outstanding technical requirements.

Within one week, you’ll receive an e-mail detailing the required amendments. When these have been addressed, you’ll receive a formal acceptance letter and your manuscript will be scheduled for publication.

An invoice will be generated when your article is formally accepted. Please note, if your institution has a publishing partnership with PLOS and your article meets the relevant criteria, all or part of your publication costs will be covered. Please make sure your user information is up-to-date by logging into Editorial Manager at Editorial Manager® and clicking the ‘Update My Information' link at the top of the page. For questions related to billing, please contact billing support.

If your institution or institutions have a press office, please notify them about your upcoming paper to help maximize its impact. If they’ll be preparing press materials, please inform our press team as soon as possible -- no later than 48 hours after receiving the formal acceptance. Your manuscript will remain under strict press embargo until 2 pm Eastern Time on the date of publication. For more information, please contact onepress@plos.org.

Kind regards,

Laura Kelly

Division Editor

PLOS One

Additional Comments (optional):

Please ensure the text in the methodology in square brackets "[insert actual score range]" is updated appropriately.

Please also ensure the Disclaimer / Publisher's Note is removed, as this pertains to a different publisher and not PLOS One.

Reviewers' comments:

Reviewer's Responses to Questions

**Comments to the Author**

1. If the authors have adequately addressed your comments raised in a previous round of review and you feel that this manuscript is now acceptable for publication, you may indicate that here to bypass the “Comments to the Author” section, enter your conflict of interest statement in the “Confidential to Editor” section, and submit your "Accept" recommendation.

Reviewer #2: All comments have been addressed

**********

2. Is the manuscript technically sound, and do the data support the conclusions?

The manuscript must describe a technically sound piece of scientific research with data that supports the conclusions. Experiments must have been conducted rigorously, with appropriate controls, replication, and sample sizes. The conclusions must be drawn appropriately based on the data presented.

Reviewer #2: Yes

**********

3. Has the statistical analysis been performed appropriately and rigorously?

Reviewer #2: Yes

**********

4. Have the authors made all data underlying the findings in their manuscript fully available?

The PLOS Data policy requires authors to make all data underlying the findings described in their manuscript fully available without restriction, with rare exception (please refer to the Data Availability Statement in the manuscript PDF file). The data should be provided as part of the manuscript or its supporting information, or deposited to a public repository. For example, in addition to summary statistics, the data points behind means, medians and variance measures should be available. If there are restrictions on publicly sharing data—e.g. participant privacy or use of data from a third party—those must be specified.

Reviewer #2: Yes

**********

5. Is the manuscript presented in an intelligible fashion and written in standard English?

PLOS ONE does not copyedit accepted manuscripts, so the language in submitted articles must be clear, correct, and unambiguous. Any typographical or grammatical errors should be corrected at revision, so please note any specific errors here.

Reviewer #2: Yes

**********

6. Review Comments to the Author

Please use the space provided to explain your answers to the questions above. You may also include additional comments for the author, including concerns about dual publication, research ethics, or publication ethics. (Please upload your review as an attachment if it exceeds 20,000 characters)

Reviewer #2: Thank you for addressing my comments. The manuscript has improved significantly and I recommend its acceptance.

**********

7. PLOS authors have the option to publish the peer review history of their article (what does this mean?). If published, this will include your full peer review and any attached files.

If you choose “no”, your identity will remain anonymous but your review may still be made public.

**Do you want your identity to be public for this peer review?**  For information about this choice, including consent withdrawal, please see our Privacy Policy.

Reviewer #2: **Yes:**  Abdullahi Yusuf

**********

# Editor report: Acceptance letter

**DOI:** 10.1371/journal.pone.0344618.r004

**Authors:** Wei Lun Wong, Laura Kelly

PONE-D-26-07893R1

PLOS One

Dear Dr. IRGATOĞLU,

I'm pleased to inform you that your manuscript has been deemed suitable for publication in PLOS One. Congratulations! Your manuscript is now being handed over to our production team.

At this stage, our production department will prepare your paper for publication. This includes ensuring the following:

* All references, tables, and figures are properly cited

* All relevant supporting information is included in the manuscript submission,

* There are no issues that prevent the paper from being properly typeset

You will receive further instructions from the production team, including instructions on how to review your proof when it is ready. Please keep in mind that we are working through a large volume of accepted articles, so please give us a few days to review your paper and let you know the next and final steps.

Lastly, if your institution or institutions have a press office, please let them know about your upcoming paper now to help maximize its impact. If they'll be preparing press materials, please inform our press team within the next 48 hours. Your manuscript will remain under strict press embargo until 2 pm Eastern Time on the date of publication. For more information, please contact onepress@plos.org.

You will receive an invoice from PLOS for your publication fee after your manuscript has reached the completed accept phase. If you receive an email requesting payment before acceptance or for any other service, this may be a phishing scheme. Learn how to identify phishing emails and protect your accounts at https://explore.plos.org/phishing.

If we can help with anything else, please email us at customercare@plos.org.

Thank you for submitting your work to PLOS ONE and supporting open access.

Kind regards,

PLOS ONE Editorial Office Staff

on behalf of

Dr. Laura Hannah Kelly

Staff Editor

PLOS One
